# Innovations in Nanoemulsion Technology: Enhancing Drug Delivery for Oral, Parenteral, and Ophthalmic Applications

**DOI:** 10.3390/pharmaceutics16101333

**Published:** 2024-10-17

**Authors:** Shery Jacob, Fathima Sheik Kather, Sai H. S. Boddu, Jigar Shah, Anroop B. Nair

**Affiliations:** 1 Department of Pharmaceutical Sciences, College of Pharmacy, Gulf Medical University, Ajman 4184, United Arab Emirates; fathima.sheik@gmu.ac.ae; 2 Department of Pharmaceutical Sciences, College of Pharmacy and Health Sciences, Ajman University, Ajman 346, United Arab Emirates; s.boddu@ajman.ac.ae; 3 Center of Medical and Bio-Allied Health Sciences Research, Ajman University, Ajman 346, United Arab Emirates; 4 Department of Pharmaceutics, Institute of Pharmacy, Nirma University, Ahmedabad 382481, India; jigsh12@gmail.com; 5 Department of Pharmaceutical Sciences, College of Clinical Pharmacy, King Faisal University, Al-Ahsa 31982, Saudi Arabia; anair@kfu.edu.sa

**Keywords:** nanoemulsions, oral, ophthalmic, parenteral, preparation, evaluation, clinical trials, patents

## Abstract

Nanoemulsions (NEs) are submicron-sized heterogeneous biphasic liquid systems stabilized by surfactants. They are physically transparent or translucent, optically isotropic, and kinetically stable, with droplet sizes ranging from 20 to 500 nm. Their unique properties, such as high surface area, small droplet size, enhanced bioavailability, excellent physical stability, and rapid digestibility, make them ideal for encapsulating various active substances. This review focuses on recent advancements, future prospects, and challenges in the field of NEs, particularly in oral, parenteral, and ophthalmic delivery. It also discusses recent clinical trials and patents. Different types of in vitro and in vivo NE characterization techniques are summarized. High-energy and low-energy preparation methods are briefly described with diagrams. Formulation considerations and commonly used excipients for oral, ocular, and ophthalmic drug delivery are presented. The review emphasizes the need for new functional excipients to improve the permeation of large molecular weight unstable proteins, oligonucleotides, and hydrophilic drugs to advance drug delivery rapidly.

## 1. Introduction

Nanoemulsions (NEs) are submicron (20–500 nm) isotropic colloidal dispersion systems that appear optically clear or translucent. They generally consist of aqueous and oil phases, with a surfactant serving as the primary emulsifying agent, often supplemented by intermediate-length alkanols as auxiliary emulsifiers or cosurfactants, and may occasionally contain an electrolyte [1]. NEs represent a thermodynamically unstable system that requires external energy input to transform into a colloidal dispersion. Nevertheless, they exhibit kinetic stability against gravitational separation and aggregation over extended periods [2].

Microemulsions constitute a thermodynamically stable system, typically characterized by a high surfactant-to-oil ratio [3,4]. They are often spontaneously formed by mixing all components (oil, water, and surfactant–cosurfactant mixture) together at a specific temperature, without the need for external energy input [5]. These systems can adopt various structures, including water-continuous, oil-continuous, or bicontinuous, contingent on the concentrations, nature, and arrangements of the molecules involved. Consequently, microemulsions may exhibit spherical, lamellar, or bicontinuous structures. Despite their thermodynamic stability, microemulsions may become unstable if their components undergo chemical changes or if external stress alters the conditions to a point where the system cannot remain thermodynamically stable. The small radius of the formed droplets, coupled with the high interfacial tension (γ) of the system, results in a significant Laplace pressure (ΔPL = 2γ/r), which favors a reduction in the interfacial area. Consequently, the droplets assume a spherical shape, as spheres possess the lowest surface free energy per unit volume [6]. Emulsification occurs when the Gibbs surface free energy between two immiscible liquids is minimized, as calculated by the expression ΔG = ΔAγ − TΔS [7]. This reduction is achieved by adding emulsifiers, which lower γ between the two fluids, thereby compensating for the high surface area resulting from particle size reduction and the very small entropy of the nanosystem (TΔS). The type of microemulsion formed will change according to the surfactant, cosurfactant, oil, and aqueous phases used and the properties they each hold. Selection of appropriate microemulsion components does not necessarily adhere to strict guidelines, but decisions based on empirical observations can be followed. The complete benefits of microemulsions as drug delivery carriers are not realized due to the lack of availability of a wide range of pharmaceutically acceptable surfactants, cosurfactants, oils, solvents, and other materials [8]. The surfactant(s) selected for a specific oil must have the correct hydrophilic-lipophilic balance (HLB) to achieve the appropriate curve at the interfacial zone for the intended kind of microemulsion. This balance is crucial for significantly reducing γ to aid in dispersion during microemulsion preparation and for forming a highly flexible film. The conceptual framework that relates molecular parameters to surfactant microstructures or film curvatures can be described using a critical packing ratio (CPP), expressed as V/al, wherein V represents the volume of the hydrocarbon core, a is the effective area of the polar head group, and l is the maximum hydrocarbon tail length of the surfactant [9]. Oil-in-water (O/W) microemulsions are preferred in the system if the surfactant’s polar region is much larger than the hydrophobic region, with CPP varying from 0 to 1, and the interface spontaneously curves to water (positive curvature). When the CPP is more than 1, water-in-oil (W/O) microemulsions are created, and the interface curves negatively (negative curvature) [10]. When the HLB is balanced and CPP is 0, there is virtually zero curvature, and depending on the film’s rigidity, either bicontinuous or lamellar structures are formed [11]. The incorporation of the oil and cosurfactant, along with the hydration of the surfactant head group to form the surfactant film, also affects the packing and curvature. The significant decrease in particle size leads to an increase in surface area, thereby enhancing solubility, dissolution, and bioavailability. NEs are prone to time-bound physical instability problems that coarse emulsions may face, such as sedimentation, creaming, Ostwald ripening, phase separation, and coalescence [8].

Additionally, NEs may undergo chemical and biochemical degradation during storage, resulting in organoleptic changes, and most frequently, lipid oxidation [12]. NE stability is also influenced by factors such as temperature, pH, and ionic strength [13]. Additionally, it is clear that the inactive ingredients added to the formulation can significantly alter the chemical characteristics, stability, and in vitro release properties of the NEs [14]. NEs represent lipid-based nano-vesicular carriers that enhance the bioavailability and solubility of hydrophobic drugs and bioactive nutritional components, including flavonoids, non-flavonoids, and carotenoids [15]. The chemical stability of NE can be assessed using various spectral analyses, chromatographic or electrophoretic methods.

## 2. Theories of Emulsification

Interfacial or mixed film theories explain that NE formation and its long-term stability involve different interfaces and molecular films. Different surfactants or mixtures of surfactants can form more complex interfacial films, contributing to kinetic stability, where the rate of separation is significantly slowed down over practical timescales [16]. Stability is influenced by factors such as the elasticity of the interfacial film, the density difference between the oil and water phases, and the size distribution of the droplets. The equation below, relating two-dimensional dispersion pressure (Π) due to the Laplace pressure effect across the curved interface to γ_i_, explains how γ influences the pressure across a curved interface, which is essential for understanding the stability of emulsions [17].

$$\pi =\gamma_{i}\left(\frac{1}{R_{1}}+\frac{1}{R_{2}}\right)$$

where R_1_ and R_2_ are the principal radii of curvature of the interface.

Solubilization theory states that in the presence of sufficient surfactant concentration, resulting in the formation of normal or reverse micelles, oil molecules can be solubilized in their hydrophobic core, leading to the formation of nano-sized droplets [18]. In general, droplet size and distribution theory focuses on factors such as the concentration and type of surfactant, the method of emulsification, and the energy input that influences the size and distribution of droplets in an NE, which are critical for their stability and properties. According to Bancroft’s rule, the continuous phase of an emulsion is determined by the phase in which the surfactant is more soluble. Surfactant packing parameter theory explains the formation of NEs based on the molecular geometry or CPP of surfactants. Phase behavioral theory examines the phase behavior of surfactant, oil, and water mixtures to predict the conditions under which NEs form. In addition, NEs form in regions where the system transitions from a single phase to multiple phases, facilitated by the surfactant’s potential to stabilize the interface between the oil and water phases. Thermodynamic stability theory suggests that the stability of NE systems is due to the reduction in the surface free energy of the formed spherical globules, while kinetic stability theory proposes that surfactants adsorb onto the surface of the generated emulsion droplets, preventing coalescence and Ostwald ripening, thereby contributing to the overall stability of the system [19,20]. These theories provide a comprehensive understanding of how NEs are formed and stabilized, highlighting the importance of surfactants, γ, energy input, and phase behavior in the process.

## 3. Formulation Components

### 3.1. Oils

The composition and maximum allowable concentrations of FDA-approved excipients used for the creation of NEs are based on the intended use and administration route. Formulating NEs involves using only components that are deemed safe and are generally recognized as safe, which include oils, surfactants, emulsifying agents, polymers, viscosity and density modifiers, and ripening inhibitors [21]. The chemical stability of NEs can be enhanced by several methods, including antioxidants or complexing agents, adjusting the characteristics of the interfacial film such as its strength, flexibility, charge, thickness, and chemical inertness, and protecting them from various environmental stress factors, viz., pH, oxygen, and temperature [22]. Typically, NEs contain 5 to 20% dispersed phase in O/W emulsion, though the lipid concentration can be nearly 70% [23]. Because of decreased optical and mechanical characteristics, lipid-based interfacial films tend to be thick, brittle, and translucent. Currently, the oil phase mainly includes long-chain, medium-chain, and short-chain triglycerides, ethyl oleate, oleic acid, and vitamin E (tocopherol), either alone or in combination [24]. Various synthetic lipids, namely Caproyl 90, triacetin, isopropyl myristate, oleic acid, palm oil esters, isopropyl palmitate, Labrafil M1944CS, Maisine 35-1, Miglyol 812, Captex 200, Captex 355, and Captex 8000, are frequently utilized in NE production [25]. The choice of oil involves balancing its ability to solubilize the drug with its capacity to form a microemulsion. When developing NEs with natural oils, an HLB value above 10 usually results in O/W NEs, while a value below 10 typically produces W/O NEs [26].

### 3.2. Emulsifying Agents

Emulsifying agents prevent globule coalescence by either reducing γ for thermodynamic stabilization, forming a rigid interfacial film as a mechanical barrier, or forming an electrical double layer to inhibit particle approach. They often adsorb quickly on the oil–water interface, leading to either combined electro-steric stabilization or electrostatic stabilization. Common emulsifiers include lecithin (phosphatidylcholine), polyoxyl 35 castor oil (Cremophor EL), sodium deoxycholate (bile salt), and Solutol HS-15 (polyoxyethylene-660-hydroxy stearate). When choosing an emulsifier, it is crucial to consider both the CPP and the HLB. Selecting the appropriate emulsifier and its concentration is crucial for forming a stable, coherent, and flexible film at low concentrations, which provides a barrier to coalescence and is fundamental to the emulsification process [27]. Emulsifying agents are categorized by the type of film they form at the interface: surface-active agents produce monomolecular films, semi-synthetic and natural hydrocolloid emulsifiers produce multimolecular films, and finely divided solid particles form particulate films. Research has shown that different proteins can act as efficient emulsifiers, especially in O/W NEs. They stabilize the interface by combining steric hindrance with electrostatic repulsion [28]. However, because of their electrical characteristics, proteins’ ability to stabilize emulsions is affected by variations in pH and ionic strength. When proteins are concentrated in salt or close to their isoelectric points, they tend to cluster, which decreases electrostatic repulsion [29]. In contrast, polysaccharides are not well-suited for NEs due to their tendency to absorb water and their high solubility, which results in poor moisture barrier properties for liquid products [30].

### 3.3. Surfactants and Cosurfactants

Surfactants adsorb at the interface between the dispersion medium and droplets to form a monomolecular flexible film. This leads to a decrease in γ and surface free energy, hence decreasing the tendency for coalescence of droplets, thereby achieving the stability of NEs [31]. Adding a cosurfactant or auxiliary emulsifying agents such as medium- or short-chain alcohols as well as polyols can function as a thickening agent and help stabilize the emulsion. These additives reduce γ and enhance fluidity at the interface in NEs. Enhancing the mobility of hydrocarbon tails increases miscibility and improves oil diffusion in this region. The preparation of NEs is significantly influenced by the selection and concentration of an appropriate surfactant to facilitate quick adsorption and stabilization of formed nanoscale droplets, reaching pressures of 10 to 100 atm. The flexibility of the interfacial film contributed by the surfactant is also important, as it can reform rapidly if broken or disturbed. Finally, the dispersed phase must continue to be extremely insoluble in the dispersion medium in order to prevent Ostwald ripening. A recent publication describes a method by which stable NEs are generated via a nanopore system without the use of surfactants. In this method, an oil solution is pumped at a controlled flow rate through a membrane with 100 nm pores into an aqueous solution while ultrasound is applied [32]. Non-ionic surfactants offer numerous benefits, including low toxicity, non-irritancy, and reduced aggregation through hydration, temperature swings, and steric hindrances [33]. Frequently used non-ionic surfactants encompass polyoxyethylene sorbitan monolaurate 20, 40, 60, and 80 (Tweens), sorbitan monolaurate 20, 40, 60, and 80 (Spans), polyglycerol esters of fatty acids, polyoxyethylene ether surfactants (Brij 97), and sucrose monopalmitate. Conversely, anionic surfactants (e.g., sodium lauryl sulfate, sodium dodecyl sulfate) or cationic surfactants (e.g., β-lactoglobulin) avoid droplet accumulation through electrostatic repulsive forces but can cause irritation at high concentrations, limiting their use in products requiring high surfactant levels. Zwitterionic surfactants (e.g., lecithin) offer high surface activity, biodegradability, foam stability, low irritation, low toxicity, a low critical micelle concentration, and high water solubility, although they are more expensive than other surfactants [34,35]. A liquid crystal emulsion is a novel emulsion type where emulsifier molecules form a long-range ordered and short-range disordered lamellar liquid crystal at the oil/water interface. A combination of hydrogenated lecithin and phytosterol emulsifiers was used to create a liquid crystal NE stabilized with a microfluidizer [36]. Simulations showed that this combination enhanced the elasticity of the bilayer structure, supporting the existence of larger curvature at the interface between the lipid and aqueous phases. The cosurfactant aids in preserving the emulsifier film’s flexibility, enabling it to deform smoothly around each droplet by lessening interactions between polar head groups and hydrocarbon chains. Short to medium-chain alcohols are frequently used as effective cosurfactants.

### 3.4. Density and Viscosity Modifiers

Materials like polysaccharides act as gelling or thickening agents in the aqueous phase (e.g., agar, pectin, xanthan gum, sodium alginate, tragacanth) and proteins (e.g., casein, albumin) enhance emulsion stability by minimizing droplet movement and also impart desirable textural attributes, including gel strength, creaminess, thickness, and richness [22]. For maximum stability, it is essential to attain an optimal zeta potential and viscosity in the system [37]. To prevent creaming in NEs with a lower-density oil phase, density modifiers or ripening inhibitors such as sucrose acetate isobutyrate are added [38]. Highly hydrophobic molecules, such as long-chain triglycerides with low-water solubility (e.g., corn oil, grape seed oil, palm oil, rapeseed oil, and sunflower oil), are added to the dispersed phase of NEs to prevent droplet growth caused by Ostwald ripening, which can interfere with the mixing process. Typically, ripening inhibitors are used in the production of O/W NEs containing highly water-soluble oil phases, such as essential oils and flavors [24].

## 4. Preparation Methods

NEs generally require energy-intensive methods like high-pressure homogenization (HPH), ultrasonication, rotor-stator emulsification (RSE), microfluidics, high-shear mixing, and membrane methods. Alternatively, they can be produced using low-energy techniques like phase inversion composition, d-phase emulsification, phase inversion temperature (PIT), and self-nano emulsification. Low-energy techniques are preferred for their lower energy consumption, greater efficiency, and lack of need for sophisticated instruments. Nevertheless, high-energy methods often require less surfactant mixture than low-energy methods, making them more suitable for nutritional preparations [39].

### 4.1. High Energy Methods

These are top-down methods that utilize high-shear disruptive forces like hydraulic shear, turbulence, and cavitation to break up large globules into nano-sized emulsion droplets [40]. Despite shear forces being applied over a short period of time, the intense energy and elevated temperature produced during the process can lead to the decomposition of thermolabile compounds such as proteins, peptides, and enzymes [41]. By employing high-energy methods along with suitable formulation components, greater control over globule size, rheology, color, and stability of NEs can be achieved [22].

#### 4.1.1. High Pressure Homogenization (HPH)

**Figure 1:**
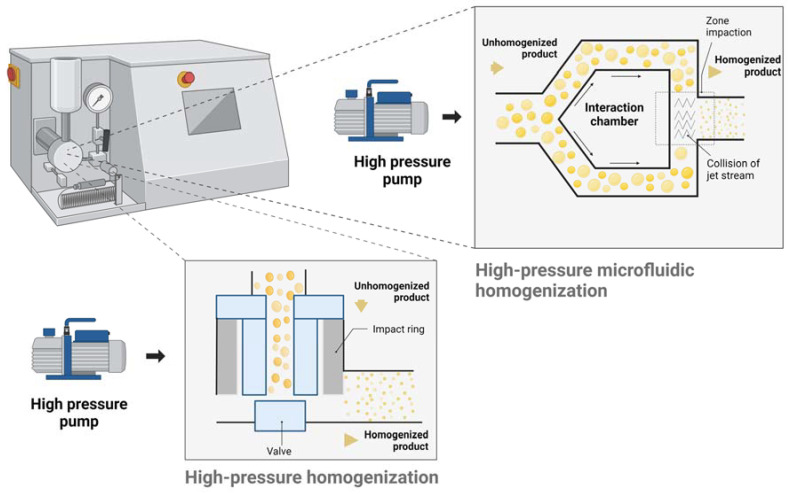
Schematic illustration of high-pressure homogenization and microfluidization process (created with BioRender.com, accessed on 23 September 2024).

In this procedure, coarse pre-emulsions are allowed to pass through at high pressure (500–5000 psi) through the narrow valve situated downstream by means of a piston pump to generate a NE with a narrow size distribution (Figure 1) [42]. Throughout this process, hydraulic shear and intense turbulence reduce the size of macro-sized droplets to minute ones, repeating this multiple times until the NEs are successfully formed [43,44]. In the optimization of high-pressure homogenization, three key process variables are considered: the number of pass cycles, applied pressure, and process temperature, while the formulation variables typically include emulsifier type and concentration [45]. Researchers utilized HPH to create NEs, reporting a particle size of 89.7 ± 27.7 nm, a polydispersity index (PDI) of 0.226 ± 0.021, and a zeta potential of −12.54 ± 1.67 mV [46]. Ultra-HPH was utilized to generate soybean protein NEs stabilized by soybean protein isolate, β-conglycinin, and glycinin [47]. These NEs demonstrated stability under diverse conditions, including different ionic concentrations, pH, temperature, and periods of storage. It was proposed that the enhanced stability of the NEs could be related to the secondary structure of stabilizers, shaped by the ultra-HPH process.

The industrial scale-up of HPH for NE preparation faces numerous challenges due to high energy requirements during long process times and the need for specialized equipment. In addition, operating machinery at high pressures or through numerous passes for extended periods leads to wear and tear on machine parts. Additionally, batch processing methods do not align well with the continuous flow and low energy consumption requirements of industrial settings. Furthermore, the intense nature of HPH (20–100 MPa) may result in undesirable temperature increases, potentially affecting drug product quality. Therefore, reducing energy requirements is crucial for lowering the processing costs of emulsification and enhancing the sustainability of NE production. The development of pepper NEs involved using a high-speed homogenizer immediately followed by HPH. The nature and amount of surfactants, the HLB, the ratio of lipid to aqueous phase, the ratio of surfactant to oil, and the makeup of the pepper extract in the NE were among the many formulation and process characteristics that were considered [48]. The research investigated the use of HPH and ultrasonication in combination to create stable NEs. A mixture comprising 15% (*w*/*w*) O/W, along with 4.5% (*w*/*w*) of Tween 80 and Span 80 combination (1:1 *w*/*w*), was utilized. Applying ultrasonication and HPH together at low and medium energy levels resulted in NEs with reduced average particle size and, in most cases, enhanced stability compared to those produced using either ultrasonication or HPH alone at higher energy levels. Additionally, it was observed that ultrasonication followed by HPH exhibited greater efficacy in NE preparation [49]. When the phase inversion composition approach (low-energy emulsification) and the high-energy method were compared, the globule size produced by each method did not significantly vary [50]. However, the PDI showed significant variation (*p* < 0.001). Additionally, over 60% of the drug was released within the first two hours of the dissolution testing, indicating a highly significant difference (*p* < 0.001) in drug release between formulations generated by each approach.

#### 4.1.2. Ultrasonic Homogenization

**Figure 2:**
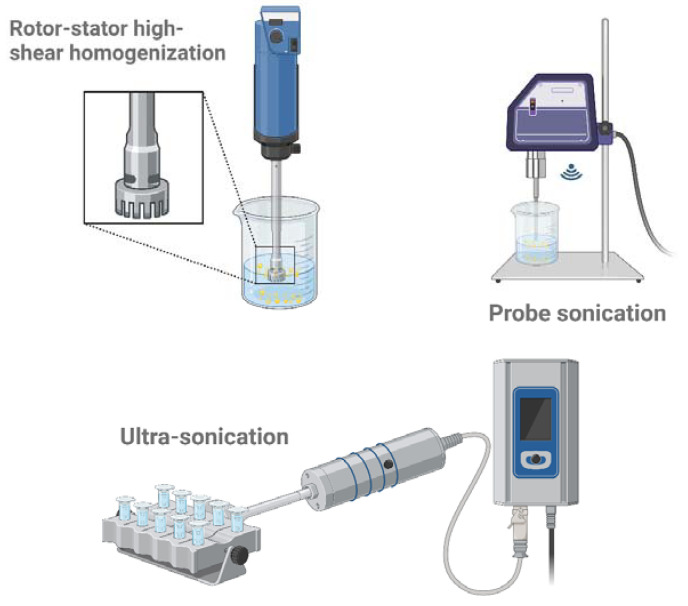
Schematic illustration of rotor-stator high-shear homogenization, probe sonication, and ultrasonication (created with BioRender.com, accessed on 23 September 2024).

Ultrasound emulsification is a technique that utilizes acoustic cavitation forces and waves (Figure 2). This process involves creating and collapsing microbubbles at the interface by placing a sonication probe inside the coarse emulsion [51]. The process of acoustic emulsification was described as an integration of dispersed phases into aqueous media, induced by Rayleigh–Taylor instability caused by ultrasonic waves at the media surface, leading to cavitation [52]. In batch ultrasonic systems, an ultrasonic probe is submerged into a vessel containing oil and water phases, where it applies intense ultrasonic waves (20 kHz to 100 MHz) to produce small droplets (Figure 2). This method boasts advantages such as easy operation, high energy efficiency, and cost-effectiveness, necessitating low emulsifier concentrations, showcasing strong dispersion stability, and minimizing the likelihood of microbial contamination. However, potential drawbacks include prolonged processing times, the necessity for a minimum emulsion volume, and sonication energy dictated by the surface area of the sonicator tip. The cavitation process and, consequently, the size and PDI of NE droplets are primarily governed by major parameters such as frequency, time, power, and the constitution of the oil as well as aqueous phases. Several researchers have acknowledged the various advantages of ultrasonic emulsification over conventional methods, including the production of emulsions with smaller particle sizes and narrower distributions, enhanced stability, reduced surfactant requirements, energy efficiency, lower production costs, decreased risk of contamination, and ease of operation, cleaning, and apparatus control [53]. NEs have been produced using sonication methods with dispersed phase concentrations ranging widely, from approximately 0.25% to 25%. It has been reported that lowering the oil concentration and/or increasing the surfactant concentration diminishes the droplet coalescence rate during sonication, producing finer droplets [54]. Polysaccharides and proteins have also been found to be applicable in the formulation of ultrasound-induced NEs, serving as emulsifiers, thickening agents, or gelling agents [55]. The temperature elevation typically reduces droplet disruption and yields smaller droplets in an emulsion, such as viscosity, γ, and Laplace pressure. However, elevated temperatures may also accelerate droplet recoalescence due to diminished emulsifier functionality, which increases droplet size [56].

NEs of β-carotene were created using a mixed surfactant approach via the ultrasonic homogenization method, without the use of any cosurfactant [57]. In a separate investigation, NEs containing lutein, a natural antioxidant, were formulated using the ultrasonic homogenization technique [58]. The components and preparation parameters were fine-tuned through response surface methodology to determine the optimal conditions, which were subsequently validated. The formulation comprised 1.5 wt% medium-chain triglyceride (MCT) and 11 wt% Tween 80, with ultrasonic homogenization conducted for 8.5 min. The resultant NE exhibited an average particle size of around 100 nm. Furthermore, enhancements in solubility and stability were observed, with the addition of propylene glycol notably augmenting stability. To enhance the efficiency of the homogenization process, reduce energy requirements, and consequently lower costs, NEs are produced by integrating various techniques, such as HPH with ultrasonic homogenization [49,59].

#### 4.1.3. Rotor-Stator Emulsification (RSE)

Rotor-stator equipment offers numerous advantages, including straightforward installation, less financial investment, compatibility with viscous systems, and the ability to prepare large volumes of emulsion. The rotating element, equipped with blades or teeth, and the stationary component of the axially fitted stator create a narrow gap or shearing zone between them, generating high shear forces during rotation, resulting in intense mixing, emulsification, dispersion, or homogenization of fluids or substances passing through the equipment (Figure 2). Rotor-stator systems can operate either discontinuously utilizing quasi-continuous production or continuously with colloid mills equipped with gear-rim dispersing machines, smooth or toothed rotors and stators, and intensive mixers [60]. The crucial product parameters concerning industrial emulsification processes include viscosity ratio, viscosity and fraction of the dispersed phase, viscosity of the continuous phase, emulsifier adsorption rate, product stress, and retention time in the dispersing unit. Colloid mills are utilized in producing emulsions characterized by moderate (20 mPa s) to high viscosities (8000 mPa s). A rotor-stator system coupled with a high-shear mixer was employed to fabricate NE droplets with an average size of 87 nm, consisting of 10% algae oil, 2.8% Tween 40, and water. This was achieved using a rotor-stator system coupled with a high-shear mixer [61]. A central composite rotatable design was used to examine the effects of processing variables on the physical-chemical characteristics of a thymol NE, employing a high-speed rotor/stator-type homogenizer [62]. The optimized formulation, consisting of 1.0% thymol, 4.63% Tween 80, a rotor speed of 6600 rpm, and a rotation time of 3.13 min, resulted in NE droplets with an average size of 68.47 nm, a PDI of 0.112, and a zeta potential of −11.00 mV. These enhanced NEs maintained stable droplet sizes (<200 nm) for over 45 days when kept at 4 °C under various conditions (0.5% NaCl, 1.0% NaCl, pH 4.5, and pH 7.5). The observed properties of the nanodroplets highlight the effectiveness of high-speed homogenizers in NE production. When choosing an emulsification method, considering emulsion viscosity is critical. High-pressure systems are suitable for emulsions requiring very low viscosity, while rotor-stator and disc-disc systems are better for higher viscosities, achieving smaller droplet diameters as well as narrow droplet size distributions. It is important to note that high-pressure systems can potentially harm various pharmaceutical or dietary supplements, including vitamins, as well as the emulsifier itself.

#### 4.1.4. High-Pressure Microfluidic Homogenization

The microfluidizer, or high-pressure microfluidic homogenizer, functions as a stationary mechanical agitator, incorporating a high-pressure generator or pneumatic pump. During its operation, the fluid being processed flows through a converging section known as the valve space before expanding to produce NE. Researchers used microfluidic technology to prepare multivesicular liposomes (MVLs) for delivering gastrodin (GAS), a water-soluble drug, across the blood-brain barrier [63]. They optimized the formulations and preparation parameters for gastrodin multivesicular liposomes (GAS-MVLs). The microfluidic method produced much smaller MVLs (2.09 ± 0.17 μm) compared to the traditional method, which typically yielded particles larger than 10 μm. The key factors influencing GAS-MVL preparation were the flow rate ratio and total flow rate, which affected the size and encapsulation efficiency. Under optimal conditions, the emulsions dispersed uniformly, resulting in dense GAS-MVL structures. The key differences between microfluidizers and traditional high-pressure homogenizers are found in the type of pump used, the achievable pressures, and whether a nozzle is present. During the microfluidization procedure, an emulsion with coarse particles is normally injected into the system through the intake reservoir and driven by a high-pressure pneumatic pump into an interaction chamber (IXC) of the Z or Y type (Figure 1). Following that, the fluid is driven at high speeds and simultaneously impacts from two opposing channels within the IXC. To release extra heat, the sample leaves through a cooling coil outlet or heat exchanger. Small emulsion droplets with a homogeneous size distribution are produced using this process [64]. In the IXC, cavitation stress and the two main flow types (turbulent and laminar) lead to the creation of NEs [65]. Because of the considerable turbulence generated by the equipment, the Y-type IXC featuring narrow microchannels (75 µm) is typically paired with an auxiliary processing mode, which invariably consists of a Z-type chamber [66,67]. Microfluidic devices have proven effective in bulk production of NEs with specific sizes [68]. Studies have indicated that this homogenizer surpasses HPH in efficiency when generating NEs in a single pass under equivalent operating pressure conditions [43,45]. The HPH and microfluidizer methods, both employing high-pressure positive displacement pumps to create NEs within the range of 30 MPa to 120 MPa, exhibit considerable differences in specific device designs [43].

Using microfluidization, high-oleic palm oil (HOPO) in water NE was produced. Based on a surface response design methodology, the process included oil concentrations from 1% to 20% *w*/*w*, mixed with whey (1% to 20% *w*/*w*) and Tween 20 at a 1:1 *w*/*w* ratio, and water [69]. The resulting NEs exhibited average droplet sizes ranging from 163 to 2268 nm, PDI values between 0.2 and 1, and zeta potential values spanning from −29.7 to −47.2 mV. Temperature during storage had a major impact on viscosity; after four days at 19 °C, it increased by about six times in comparison to the viscosity of the fresh sample. Temperatures exceeding 60 °C led to aggregation and destabilization of the palm oil droplets due to whey denaturation. In a separate study by the same author, the average droplet size and zeta potential were found to significantly impact the stability of HOPO NE [70]. Various research groups have prepared NEs of different particle sizes using microfluidization techniques to obtain the smallest stable NE with an average particle diameter of 148 nm [71], 160 nm [72], and 275.5 nm [73]. NEs with rosemary essential oil and surfactant blends were created using microfluidization and analyzed using response surface methodology [74]. An optimal HLB value of 10.5 was identified, along with an R value of 1, resulting in the smallest volumetric mean diameter (dv = 2.88 nm) of nanodroplets. Notably, NEs formulated with 5 and 7.5 g/100 g of rosemary essential oil demonstrated enhanced physical stability, suggesting promising applications as delivery systems.

### 4.2. Low Energy Methods

Low-energy methods for producing NEs utilize the system’s inherent chemical energy instead of relying on external mechanical forces. These methods exploit the phase behavior of the components, often using spontaneous emulsification techniques such as the emulsion inversion point method and the PIT method. Another technique is the solvent displacement method, where an oil phase containing water-miscible organic solvent is added and mixed into water, leading to the formation of nano-sized droplets as the solvent diffuses. These methods are advantageous due to their simplicity, cost-effectiveness, and lower energy requirements in comparison to high-energy methods like ultrasonication or HPH.

#### 4.2.1. Phase Inversion Composition (PIC)

**Figure 3:**
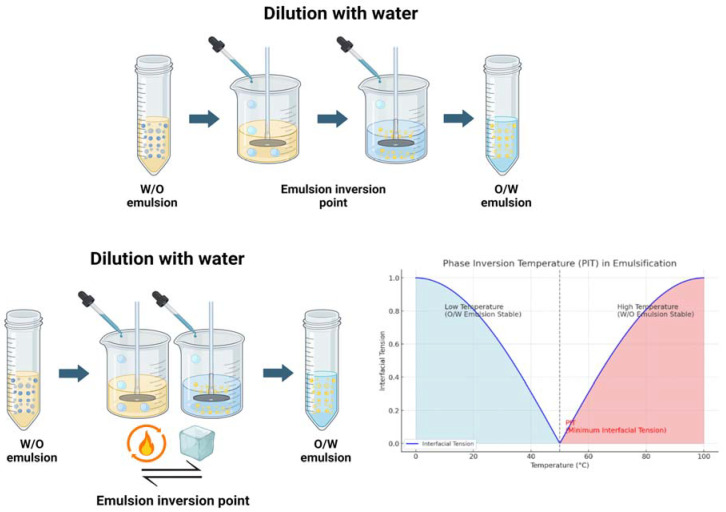
Schematic illustration of phase inversion composition and phase inversion temperature (created with BioRender.com, accessed on 23 September 2024).

In the PIC method, or emulsion inversion point method, initially formed W/O or O/W emulsions are inverted by changing the composition or conditions of the system, typically through a modification in the surfactant curvature [75]. The technique typically involves creating a primary emulsion system, which is then subjected to a phase inversion process by adjusting factors such as temperature, emulsifier concentration, or the addition of co-solvents (Figure 3). At this point, it is generally agreed upon that phase transitions in the formation of NEs via the PIC method involve a change from reverse W/O to O/W systems via zero-curvature structures like bicontinuous microemulsions or lamellar liquid crystalline phases, much like those seen with the PIT method [76]. The production of nanosized droplets and the size of the resulting nano-emulsions are both influenced by the transition between lamellar liquid crystalline phases and/or bicontinuous microemulsion phases [77]. However, there’s still debate about whether the mechanisms driving this inversion are identical in both methods. Research has demonstrated that nano-emulsification can be attained in various emulsion systems through singular or combined employment of low-energy methods. These techniques consist of phase inversion, phase inversion plus self-emulsification, or self-emulsification alone. Understanding the kinetics of emulsification by phase inversion is critical, particularly since certain emulsion systems employing the phase inversion method yield nano-emulsions with broad size distributions [78]. During emulsification, the formation of transient viscous phases necessitates slower addition and sufficient shearing of the system, potentially resulting in either monomodal or bimodal size distributions of the nano-emulsions. Furthermore, it is speculated that some oil may remain outside the transient self-assembled structures, potentially leading to the formation of large and polydisperse droplets. Phase inversion composition offers several advantages in emulsion formulation, including the ability to control droplet size distribution, enhance stability, and tailor the properties of the final emulsion for specific applications.

An NE with adjustable electric charge can be obtained by altering the pH of the adjusting system using the phase inversion method. The reported method demonstrated that modifying the system’s conditions only affected the potential of the NE without significantly impacting the particle size and stability of the emulsion [79]. Contact angle measurements conducted via FTIR showcased the impact of varying electrification levels of the NE on wettability alteration. In a separate investigation, the emulsification of n-dodecane in water via the phase inversion method was successfully accomplished by altering the composition of polyoxypropylene surfactant while maintaining a constant temperature [80].

#### 4.2.2. Phase Inversion Temperature

When an emulsion changes from water distributed in oil (W/O) to oil dispersed in water (O/W), or vice versa, it reaches a crucial temperature known as the PIT (Figure 3). This transition is caused by shifts in the balance of the HLB properties of the surfactants or emulsifiers used in the system, which are affected by temperature variations [81]. In contrast to phase inversion composition methods, this technique typically produces NEs characterized by a low PDI and high emulsification efficiency [82]. During emulsification processes, the behavior of surfactants, estimated through CPP, changes depending on the temperature of the system. At low temperatures, near or below the PIT, surfactants tend to become more soluble in water, while the hydrophobic tails of the surfactants become more soluble in the oil phase as the temperature increases [83]. At lower temperatures, the arrangement of surfactant molecules at the interface leads to a positive curvature, allowing them to bulge outward towards the water phase, contributing to the stability of the O/W emulsion. At higher temperatures, the arrangement of surfactant molecules at the interface of oil droplets leads to a negative or concave curvature inward towards the oil phase, contributing to the stability of the W/O emulsion. At temperatures below the PIT, surfactant molecules are generally more soluble in the continuous phase, resulting in a reduction in the tension at the interface between the internal and external phases. At the PIT, γ between the dispersed and continuous phases reaches a minimum value [84]. The emulsion system transitions to a favorable condition for phase inversion to occur. The emulsion stabilizes in its new configuration, which may exhibit different properties such as droplet size, stability, and rheological behavior compared to the original emulsion.

Typically, NEs produced using the PIT method are of the O/W type when the system’s PIT is between 20 to 65 °C. However, if the PIT is 10 to 40 °C below the storage temperature, W/O type NEs are formed. The stability of these NEs is temperature-sensitive and can be improved with cosurfactants. The PIT method is a quick and easy way to make NEs with a narrow size distribution and small droplet sizes. To create fine oil droplets, this process entails first mixing surfactant, oil, and water at room temperature, then heating the mixture to a temperature over the PIT, and finally cooling the mixture quickly while stirring. The PIT is identified by a noticeable reduction in turbidity, indicating the presence of a bicontinuous microemulsion or lamellar structure with weak light scattering [85,86]. However, heating during the PIT process might hasten thermal deterioration and result in the loss of volatile active components, which could compromise the antimicrobial efficacy of NEs. To minimize thermal degradation, a lower PIT may be preferred, though setting it too low could cause droplet coalescence. Stable NEs with particle sizes around 100 nm have been achieved using this method, maintaining stability over 15 days, and often exhibiting droplet sizes from 20 to 100 nm and a PDI below 0.2 [82,87]. Adjustments to the PIT, such as adding inorganic salts, may be needed for optimal results [39].

Researchers conducted a comparative study to formulate NEs using both high-energy and low-energy methods [50]. They created low-energy NEs with the phase inversion composition technique, selecting formulations based on the minimum concentration of surfactant mixture (Smix) from a ternary phase diagram. In contrast, the high-energy method utilized high-pressure homogenization, optimized with Design-Expert software (Design-Expert 8.0.0.6, State-Ease Inc., Minneapolis, MN, USA). The study found no significant difference in globule sizes between the methods (*p* > 0.05), but there were significant differences in the polydispersity index (*p* < 0.001) and drug release (*p* < 0.001), with over 60% of the drug released from all formulations within the first 2 h. Another study compared three processes for producing parenteral NEs—ultrasound, microfluidizer, and premix membrane emulsification—focusing on droplet size and the integrity of the active ingredient [88]. The results showed that the premix membrane emulsification process produced monodispersed droplets measuring 335 nm, while the other methods generated NEs around 150 nm, which also contained micron-sized droplets detected by laser diffraction and optical microscopy. There were no significant differences in active degradation during emulsification among the three processes. However, at 40 °C, NEs created with the microfluidizer displayed increased molecular degradation and instability, leading to a fourfold increase in droplet size under stress conditions.

## 5. Construction of Pseudo Ternary Phase Diagram

**Figure 4:**
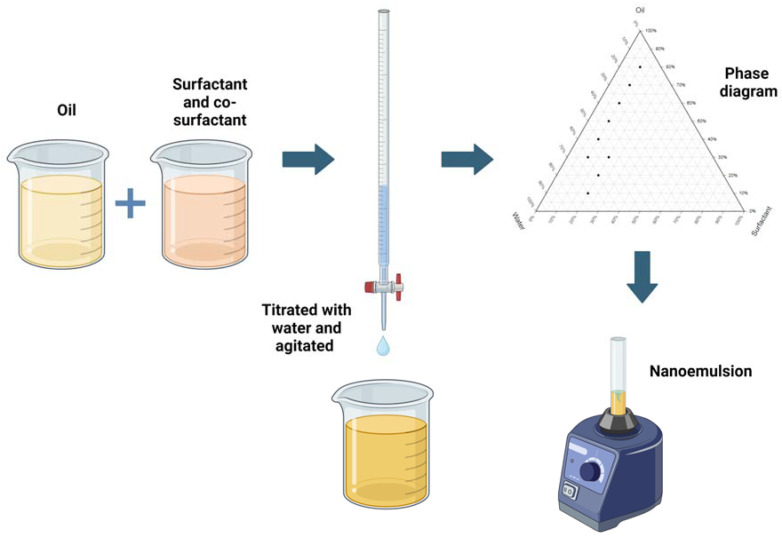
Schematic illustration of aqueous titration with pseudo ternary phase diagram (created with BioRender.com, accessed on 23 September 2024).

The aqueous titration method is a widely used technique that involves preparing thermodynamically stable NEs by blending specific ratios of oil, water, surfactant, and cosurfactant with gentle agitation [89,90]. By gradually adding the aqueous phase to the oil, surfactant, and cosurfactant mixture while continuously stirring, droplet size is progressively reduced, leading to a fine NE due to decreased γ. After selecting the appropriate components, pseudo-phase diagrams are constructed to map out different phases and identify optimal conditions for NE formation (Figure 4). The physical characteristics of the NE, such as globule diameter, stability, and clarity, are monitored and optimized by adjusting the component proportions as necessary. Ensuring the resulting NE remains thermodynamically stable is crucial, as it should not separate or degrade over time under normal storage conditions.

A study using aqueous titration was conducted to develop NEs and examine the effects of different oil phases with distinct hydrocarbon chain lengths, such as octanoic acid, Capryol 90, and ethyl oleate [9]. The phase behavior analysis demonstrated that the Smix phase played a crucial role in the nanoemulsifying capability of formulations with different oil phases. Factors such as vortexing duration, the method of incorporating the oil-Smix into the aqueous phase, the use of aqueous buffers, and the Km value significantly impacted the mean droplet size and size distribution of the NEs. Moreover, the stability profile of NEs containing fatty acid and cosurfactant showed more consistent results in terms of mean droplet size, PDI, and % transmittance at lower oil-Smix ratios compared to those containing ethyl oleate. This is due to the lower lipophilicity of fatty acid and cosurfactant compared to ethyl oleate. In a similar study, the effects of varying concentrations of sunflower oil (4–8%), vitamin B12 (5–15%), and Tween 80 (8–16%) on the physicochemical properties of vitamin B12 were investigated [91]. Optimization using a quadratic model determined that the optimal formulation comprised 9.6% Tween 80, 6.5% sunflower oil, and 13% vitamin B12. This formulation resulted in the following properties: pH of 7.24, viscosity of 17.0024 cp, turbidity of 2.19, efficiency of 51.98%, vitamin B12 concentration of 5.54 ppm, p-Anisidine index of 0.01, PDI of 0.34, and particle size of 322 nm.

## 6. Oral Nanoemulsions

NEs offer several beneficial effects in oral drug delivery, making them attractive candidates for enhancing the bioavailability and therapeutic efficacy of administered drugs. NEs can solubilize poorly water-soluble drugs, increasing their concentration in the aqueous phase and improving their absorption in the gastrointestinal tract. Dasatinib, a BCS Class II drug, was transformed into an NE using high-gravity rotating packed bed technology, which offers practical feasibility for industrial mass production [92]. NEs can encapsulate various types of drugs, including hydrophobic and hydrophilic compounds, as well as macromolecules such as proteins and peptides, offering versatility in oral drug delivery. NEs may enable the use of lower drug doses or less frequent dosing schedules, leading to reduced side effects and improved patient outcomes. A novel NE system that is oil-structured, comprising fatty acids that are phase-changeable, has been developed to provide the oral delivery of exenatide (EXT@PC/NEMs) for efficiently managing type 2 diabetes [93]. These EXT@PC/NEMs exhibit physical stability when stored at 4 °C, forming a solid core to prevent drug leakage. Upon oral administration, the deformable liquid EXT@PC/NEMs improve permeability across the intestinal epithelium via intestinal lymphatic transport, leading to pancreatic accumulation and subsequent insulin release. The study’s demonstrated increase in bioavailability suggests that orally delivering labile peptide drugs is feasible.

NEs can potentially bypass first-pass metabolism by promoting drug absorption directly into the systemic circulation through lymphatic uptake, thereby increasing the bioavailability of drugs that undergo extensive metabolism in the liver. A lipid-based formulation, such as NE, presents a promising method for delivering drugs to effectively combat infections caused by the hepatitis B virus within the lymphatic system [94]. The preparation, characterization, and bioavailability assessment of baicalin NE, targeted for lymphatic system absorption, have been demonstrated. Compared to baicalin suspension, baicalin NE exhibited a remarkable increase in AUC_0–t_ and C_max_ values, by 10.5-fold and 3.12-fold, respectively. However, when comparing rats orally administered baicalin NE with those pretreated with cycloheximide, the AUC_0–t_ and C_max_ values were reduced. Furthermore, the C_max_ value for baicalin in W/O NE was found to be 11.5-fold higher in the lymph nodes compared to the suspension-treated rats, confirming the superiority of NE for lymphatic drug transport.

NEs can be formulated to provide sustained release of drugs, prolonging their residence time in the gastrointestinal tract and maintaining therapeutic plasma levels over an extended period. A study reported the design and characterization of dried NE encapsulating tacrolimus, providing sustained release in both simulated gastric and intestinal fluids [95]. Stable NE with a neutral surface charge and an average particle diameter of 100 nm was attained based on the experimental design. Lyophilization was found to yield the highest process yield with minimal cryoprotectant, maintaining physicochemical properties post-reconstitution. Structural analysis via X-ray diffraction and differential scanning calorimetry measurements revealed that the NE shell was amorphous in colloidal suspension but crystalline upon drying, with fluidity influenced by the excipients’ structure. These findings offer insights into improving nanocarrier design, particularly for oral drug delivery applications.

NEs can protect drugs from degradation in the harsh gastrointestinal environment, such as enzymatic degradation and pH changes, thereby enhancing their stability and bioavailability. The small droplet size of NEs increases the surface area available for drug absorption, facilitating rapid and efficient uptake across the intestinal epithelium. Even though curcumin, a natural polyphenolic phytochemical, has been reported to have a number of potentially beneficial biological activities, it exhibits extremely poor aqueous solubility and low bioavailability, limiting its application [96]. A study investigated curcumin-loaded O/W NEs with 3% (*v*/*v*) MCT in the oil phase, 3% (*v*/*v*) β-conglycinin hydrolysates, and 3% (*v*/*v*) β-conglycinin-DX-40 conjugates as the emulsifiers [97]. The average droplet size of NEs stabilized by emulsifiers decreased with enzyme hydrolysis up to 12%, suggesting limited enzymolysis for optimal NE preparation. Both emulsifiers stabilized NEs and enhanced the cumulative release rate during in vitro digestion, thus increasing the overall bioaccessibility of curcumin. NEs can be designed to target specific regions of the gastrointestinal tract, such as the stomach, small intestine, or colon, by modifying their physicochemical properties and surface characteristics, or incorporating targeting ligands. This enables site-specific drug delivery, reducing systemic side effects and improving therapeutic outcomes. Extensive first-pass glucuronidation poses significant limitations on the oral bioavailability of flavonoids. An investigation was conducted to assess the potential of NEs containing pharmaceutical excipients capable of inhibiting uridine diphosphate glucuronosyltransferase (UGT) enzymes in enhancing the oral absorption of chrysin as a flavonoid model [98]. The effects of these inactive ingredients on glucuronidation were examined using tissue microsomes from both the liver and intestine, UGT1A1 enzyme expression, and UGT1A1-overexpressing HeLa cells. Among the various excipients tested, sodium oleate exhibited the most pronounced inhibitory effect on glucuronidation. NEs formulated with sodium oleate displayed a globule diameter of 83.2 nm, a surface potential of −43.7 mV, and an encapsulation efficiency of 89.5%. Pharmacokinetic analysis revealed that developed NE significantly increased chrysin’s systemic exposure by 4.3-fold and its C_max_ value by 3.5-fold, whereas the Tween80-based NE did not affect chrysin oral absorption. Consequently, it was inferred that NEs containing sodium oleate substantially enhanced the oral absorption of chrysin by selectively inhibiting first-pass glucuronidation. Table 1 depicts various types of oral NEs, including their composition, preparation techniques, and key features.

When an NE is ingested, it enters the stomach, where the highly acidic gastric juice can cause some of its constituents to chemically degrade. Lipid droplets within an NE can have their composition, size, charge, aggregation state, and interfacial properties altered by digestive enzymes and surface-active ingredients found in gastric juice, such as phospholipids and pepsin. Self-microemulsifying drug delivery systems consist of isotropic mixtures of surfactants, lipids, and co-solvents that spontaneously form kinetically stable microemulsions in the gastrointestinal tract. It is anticipated that in the small intestine, NEs undergo lipolysis, forming fatty acids. These subsequently combine to form mixed micelles with phospholipids and bile acids, which have the ability to solubilize and transfer bioactive substances across intestinal epithelial cells. It was disclosed that the emulsifier type also has a strong impact on the bioaccessibility and stability of curcumin during in vitro digestion and on cell viability [99]. Furthermore, more than 90% of the curcumin remained in the emulsion during simulated stomach digestion, most likely as a result of β-lactoglobulin’s resistance to pepsin [100]. In addition, there is a potential for the drug to precipitate in the gastrointestinal tract, depending on food composition, pH, and individual conditions.

**Table 1:** Various types of oral nanoemulsions (NEs) depicting composition, preparation techniques, characterization, and key findings.

Active(s)	Type of Emulsion	Oil/Surfactant	Technique	Characterization	In Vitro–In Vivo Findings	Reference
1,8-cineole (CIN)	O/W	CIN/Dextran-bovine serum albumin/protamine (DEX5k-BSA)	Microjet with ultraviolet irradiation	Maximum degree of conjugation (30.70%) in DEX5k-BSA was observed at pH 6. UV irradiation for 90 min was applied to enhance the stability of CIN@DEX5k-BSA. Spherical particles approximately 50 nm in size were observed in CIN@DEX5k-BSA, regardless of UV irradiation.	Both CIN@DEX5k-BSA/protamine (PTM) and CIN@DEX5k-BSA/PTM/VB12 demonstrated significantly slower release and decreased cumulative release of CIN after 24 h compared to CIN@DEX5k-BSA. The CIN@DEX5k-BSA/PTM/VB12 NE enhances the in vitro and in vivo stability of CIN, prolongs its residence time in the gastrointestinal tract, and improves permeability across intestinal epithelial cells, facilitated by Vitamin B12, thereby increasing oral bioavailability. In vivo evaluation in a mouse model of atherosclerosis demonstrated that CIN@DEX5k-BSA/PTM/VB12 significantly reduced plaque accumulation by CIN.	[101]
5-fluorouracil (FU) and Curcumin (CUR)	O/W	Medium-chain triglycerides/PEG 400, ethanol, lecithin, Tween 80	Emulsification combined with ultrasonication	Encapsulation was 97.12% for CUR and 30.81% for FU, while the loading efficiency was 3.19 ± 0.133 mg/mL for CUR and 0.68 ± 0.015 mg/mL for FU. Encapsulation efficiency for CUR/FU-NE was stable over 90 days at both 4 °C and 25 °C storage. There was no significant difference (*p* > 0.05) between the smaller and larger-scale preparations.	CUR/FU-NE formulation significantly enhanced bioavailability with AUC_(0–t)_ values increasing 8.85-fold and 8.59-fold for CUR and FU, respectively, compared to the plain drugs. In HepG2 cells, it demonstrated lower IC_50_ values and a higher tumor inhibition rate (49.29%) compared to CUR (24.84%) and FU (4.72%) alone. Ki-67 analysis confirmed its strong anti-proliferation effect, while higher IC_50_ values in L02 cells indicated lower toxicity to normal cells. Pharmacokinetic studies in rats showed significantly higher plasma concentrations of CUR and FU from CU/FU-NE. Efficacy and safety were further validated through MTT assay, confocal microscopy, and H&E staining.	[102]
Ibuprofen	O/W	Olive oil/Sucrose ester L-1695 and glycerol	D-phase emulsification	NEs containing 3% (*w*/*w*) ibuprofen produced enhanced stability with a smallest droplet size of 232.1 ± 0.451 nm, a PDI of 0.047 ± 0.014, and a zeta potential (ZP) of −31.7 ± 0.361 mV. SEM study displayed spherical droplets with uniform size distribution.	Ex vivo studies conducted employing rodent intestinal linings in the in vitro diffusion chambers revealed a 10.6-fold higher absorption profile than that of microemulsions and traditional oil-in-water systems. The NEs were found to have a two-fold increase in absorption during in vivo evaluation in a rat model; in contrast, the drug-loaded microemulsions exhibited only a 1.5-fold increase in absorption compared to the drug in an oil emulsion.	[103]
Celecoxib (CLX)	O/W	Poly(δ-decalactone) (PDL)/Poly(ethylene glycol) methyl ether (mPEG_5k_-b-PDL_3.2k_)	Nanoprecipitation method or spontaneous emulsification method	Globular size of less than 100 nm was achieved in the NEs with a high loading of CLX. Selected NE maintained a constant particle size, according to long-term (24 months) storage stability studies conducted at 4 °C.	In vitro drug release studies in stimulated gastric/intestinal fluids indicated that approximately 90% of CLX was released from the NE within 96 h. In vivo pharmacodynamic studies using a carrageenan-induced paw edema model, along with the estimation of cardiac and renal markers and histopathological assessments, confirmed the significant anti-inflammatory effects and safety of the NE compared to marketed formulations. Pharmacokinetic evaluations showed that the mean retention time of CLX was significantly higher with the NE (9.63 ± 7.98 h) than with the marketed formulation (4.13 ± 1.82 h).	[104]
Curcumin (CUR)	O/W	Medium-chain triglycerides and Soy phospholipids/Poloxamer 188	Interfacial pre-polymer deposition and spontaneous nano-emulsification	Electron microscopy evaluation of CUR-NEs revealed an average particle size of 102 ± 21 nm.	In vitro studies demonstrated that CUR-NEs exhibited significant cytotoxic effects on HSC-3 cells, systematically reducing the percentage of cells in the proliferative phases (S + G2/M) in both a dose and time-dependent manner. CUR-NEs downregulated the protein expression of the PI3K/Akt/mTOR pathway and upregulated miR-199a, which targets PI3K, in OSCC cells. Over time, CUR-NEs effectively counteracted the effects of the miR-199a inhibitor on cell proliferation and the cell cycle’s proliferative phases in OSCC cells.	[105]
Hesperetin (HN)	O/W	Triacetin/Cremophor EL35, Diethylene glycol monoethyl ether	Aqueous titration method	NEs exhibited an average particle size of 12.7 nm and a polydispersity index (PDI) of 0.072, indicating a uniform size distribution. Over 50 days of storage, the mean particle size ranged from 12.63 to 16.19 nm, drug content fluctuated between 9.49 and 9.72 mg/mL, and the PDI varied from 0.072 to 0.095. Dilution test with HCl (pH 1.2), PBS (pH 6.8), and distilled water confirmed that both particle size and PDI remained stable.	In vivo pharmacokinetic studies of NEs in male Sprague-Dawley rats demonstrated a 5.67-fold increase in AUC_0–t_ and a 2.64-fold increase in C_max_ compared to the HN suspension. Percentage lymphatic transport rose to 87.72%, and cumulative absorption increased by 1.1, 1.92, and 1.5 times in the duodenum, ileum, and jejunum, respectively. The NE significantly enhanced intestinal permeability and lymphatic transport, resulting in increased bioavailability of HN.	[106]
Leflunomide (LEF)	O/W	Clove oil/Tween^®^ 20 and PEG 400	Low energy emulsification	Chitosan-coated NEs (LEF-NE) displayed a nanometric size range between 108 ± 2.81 nm and 152 ± 3.61 nm, a PDI of less than 0.3, and ZP ranging from −16.6 ± 0.72 mV to −18.2 ± 0.81 mV. The drug content varied from 91.46 ± 0.97% to 94.83 ± 1.84%. All LEF-NEs passed thermodynamic stability tests with no phase separation. The NEs demonstrated grade A dispersibility, robustness to dilution, optical clarity, and a high cloud point.	In vivo anti-inflammatory study using male Wistar rats demonstrated that LEF-CS-NE significantly reduced the edema rate by 48.68% and decreased levels of interleukin-6, tumor necrosis factor-α, and rheumatoid factor. In comparison to the control group, LEF-CS-NE dramatically decreased the levels of glutamic pyruvic transaminase by 83.19% and glutamic oxaloacetic transaminase by 40.68% in the serum. Furthermore, histopathological evaluations of various organs showed that LEF-CS-NE had superior efficacy and safety compared to both pure and commercial LEF formulations.	[107]

### Self-Nanoemulsifying Drug Delivery Systems (SNEDDS)

Coarse NE systems are thermodynamically unstable colloids that face stability issues over time, such as flocculation, creaming, coalescence, and phase separation [41]. Their stability and in vitro release are affected by factors like temperature, pH, and the chemical properties of additives [108]. Scaling up from laboratory to large-scale production introduces challenges related to reproducibility, scalability, and cost-effectiveness. However, these challenges could be mitigated by using SNEDDS [109]. SNEDDS are isotropic mixtures of drugs, lipids, surfactants, and often hydrophilic co-solvents or cosurfactants [110]. These systems spontaneously form O/W NEs with globule sizes between 20 and 200 nm upon mild agitation in an aqueous medium [111]. This spontaneous emulsification in the gastrointestinal tract enhances solubility, dissolution, intestinal permeation, and bioavailability. Moreover, these liquid formulations can be converted into solid forms like powders or granules for oral administration [112]. The primary advantages of converting to solid dosage forms include low variability, stable and accurate dosing, high dose precision, and portability. Solidifying liquid SNEDDS also provides benefits such as preventing Ostwald ripening, delaying drug precipitation, and providing good thermodynamic stability [113]. It was reported that formed micelles during the emulsification process can help improve drug solubility by reducing the degree of supersaturation [114]. Additionally, SNEDDS provide targeted drug delivery with specific site responsiveness, offering potential protection against enzymatic breakdown [115]. SNEDDS formulations with penetrating agents and specific surfactants could enhance the bioavailability of bioactives by promoting transcellular and paracellular absorption [116].

Overall, NEs show great promise in oral drug delivery by addressing several challenges associated with conventional drug formulations, including poor solubility, low bioavailability, and rapid metabolism. Their ability to enhance drug stability, absorption, and targeted delivery makes them valuable tools for improving the efficacy and patient compliance of orally administered drugs.

## 7. Ophthalmic Nanoemulsions

The intricate anatomical structure, physiological functions, and biochemical processes of the human eye pose significant barriers to the penetration of foreign substances, including drugs. Designing and developing an effective ocular drug delivery system is a distinct challenge for formulation specialists [117]. The main goal is to bypass the eye’s protective barriers to ensure high therapeutic efficacy while avoiding any irreversible tissue damage. Many factors, such as brief retention times, restricted permeability through the corneal epithelium, fast pre-corneal clearance from high tear turnover (0.5–2.2 µL/min), nasolacrimal discharge, frequent blinking (6–15 times/min), and ineffective conjunctival absorption, frequently compromise the effectiveness of conventional eye products [118,119]. The blood-ocular barrier, including the anterior blood-aqueous and posterior blood-retinal barriers, further limits the passage of hydrophilic macromolecules into systemic circulation and prevents xenobiotics from re-entering the eye from the bloodstream [120]. The blood-aqueous humor connects the epithelial cells of the non-pigmented ciliary body epithelium and has iris blood vessels and tight junctions (zonula occludens) covered in non-fenestrated vascular endothelial cells [121]. Although drugs can quickly clear from the aqueous humor, diffusion through the densely packed vitreous humor matrix remains challenging [122]. To maintain retinal homeostasis, the blood-retinal barrier is made up of retinal pigment epithelium cells and inner retinal microvascular endothelium. These cells control the movement of nutrients, electrolytes, proteins, peptides, and osmotic water fluxes [123,124]. This barrier’s restrictive properties and the limited blood flow to the retina mean that only a small portion of systemically administered drugs reaches the posterior segment of the eye [125]. Consequently, large systemic doses are often needed to achieve therapeutic drug concentrations, which can lead to significant side effects.

As shown in a recently published review, NEs are regarded as the most promising approach to enhancing the delivery of ophthalmic drugs [126]. This extensive review elaborates on NE compositions, formulation methods, technological differences, in vitro properties, and pharmaceutical performance. The primary aim of the present review was to provide summarized information on the development of ophthalmic NEs, addressing the challenges posed by the anatomical, physiological, and biochemical barriers of the eye, as well as the inherent limitations associated with NEs.

The small droplet size of NEs facilitates better corneal permeation and residence time across the corneal tight junctions, which enhances therapeutic efficacy [127]. However, the low viscosity of NEs poses a challenge for formulation scientists who need to extend contact time on the ocular surface. To overcome this, nanocarrier formulations can be converted into in situ forming gels using stimuli-responsive polymers, which react to changes in pH, temperature, or electrolytes in the eye. This conversion increases the viscosity of the formulation, thereby prolonging contact time, enabling sustained release, improving intraocular penetration, and enhancing ocular bioavailability, which in turn improves patient compliance. Additionally, in situ gelling systems provide benefits such as ease of administration, simplified manufacturing processes, and accurate dose delivery [128].

Positively charged in situ NEs are especially favored for delivering hydrophobic drugs aimed at treating dry eye disease, ocular infections, and immune-mediated inflammatory eye conditions. Cationic NEs can prolong drug residence time by interacting electrostatically with the anionic surface of corneal mucin, thereby increasing drug bioavailability in the eye. Although combining cationic surfactants with anionic surfactants results in synergistic effects, such as enhanced surface activity and tensioactivity, they have an inherent tendency to precipitate [129]. Cationic NE represents a significant advancement in effective ophthalmic drug delivery for treating ocular infections caused by Mycobacterium tuberculosis. A rifampicin-loaded NE with specific surface modifications using either chitosan or polymyxin B was successfully developed. Cationic NEs interact electrostatically with negatively charged mucin, as demonstrated by in vitro mucoadhesion experiments. This interaction may increase the NE’s efficiency by prolonging its residence duration on the ocular surface [130].

Cetalkonium chloride, a cationic surfactant, was utilized to enhance the precorneal retention of prednisolone for the treatment of uveitis [131]. When compared to formulations without the surfactant and to free prednisolone suspension (Pred Forte^®^ 1%) in rabbits, the resulting prednisolone-loaded cationic NEs showed a delayed drug release profile and enhanced corneal flux. Clinical evaluations revealed that these cationic NEs significantly mitigated uveitis severity over three weeks compared to the drug suspension. Importantly, the formulations did not lead to an increase in intraocular pressure, did not alter pupil diameter, and exhibited no adverse effects on the cornea, retina/choroid, or iris/ciliary body, thus confirming their safety. In a separate study, high-speed homogenization and ultrasonication were used to load dorzolamide into NEs to improve ocular delivery [132]. The optimized formulation (F-Opt), developed using the Box-Behnken design, included isopropyl myristate, Tween 80, and cetyl trimethyl ammonium bromide. This formulation produced nanosized globules (336.3 nm) with a zeta potential of 32.5 mV, a PDI of 0.209, and a drug content of 99.13%. The formulation also showed acceptable pH, viscosity, refractive index, surface tension, and osmolality. In vitro mucoadhesion tests revealed better adhesion compared to plain drug and commercial eye drops. Additionally, F-Opt significantly and persistently reduced intraocular pressure in rabbits and proved safe and non-irritating in ocular Draize irritancy tests. The formulation also demonstrated physical stability at both room temperature and refrigerated conditions for one month. Research has shown that targeting amino acid transporter B (0+) (ATB0,+) and organic cation/carnitine transporter 2 with NEs can significantly increase the bioavailability of ocular medications and improve their therapeutic efficacy for eye disorders [133]. Consequently, NEs modified with stearoyl L-carnitine were carefully optimized for various pharmaceutical characteristics. The uptake and processing of these NEs in human corneal epithelial cells were extensively studied, resulting in the fine-tuning of the stearoyl L-carnitine ratio. The optimized NEs effectively targeted both transporters on the corneal epithelium, leading to enhanced ocular surface retention, improved corneal penetration, and increased drug bioavailability.

A major contributing factor to a number of systemic and ocular disorders, such as hypertensive eye problems, is oxidative stress. Researchers developed and characterized wild olive NEs, which demonstrated stability in biorelevant fluids, nanoscale size, acceptable negative zeta potential, and desired osmolality and pH [134]. Both intravitreal injection and topical ophthalmic administration of these NEs effectively reduced hypertensive morphological alterations and demonstrated a strong antioxidant effect in mice with hypertension induced by Nω-nitro-L-arginine-methyl-ester. This result was ascribed to a decrease in NADPH oxidase expression and activity in the cornea and retina.

NEs, which consist of both aqueous and oily phases, have been shown to reduce tear evaporation and enhance surface hydration, thereby addressing eye dryness more effectively than conventional eye drops [135]. It is currently understood that when an NE interacts with hyperosmolar tear fluid, it primarily dilutes the lacrimal fluid and lowers its osmolality [136]. The tear film lipid layer thickens as a result of the integration of nonpolar lipids from the oil nanodroplets. However, the interaction between the NE and tear film components, along with dilution from the aqueous phase of tears and shear stress from blinking, can compromise the physical stability of the oil nanodroplets. Nevertheless, the oil phase of the NE continues to remain on the ocular surface for a considerable amount of time (3–4 h) [137].

Dry eye disease is a common and complex condition marked by symptoms including redness, burning, eye fatigue, a sensation of a foreign body in the eye, and light sensitivity [138]. Topical applications of artificial tears and herbal drops are highly popular treatments for this disease [139]. It was demonstrated that an NE formulated with 75% water, 5% Nigella sativa seed extract, and 20% surfactant in a 1:4 ratio can significantly enhance tear production in atropine-induced dry eyes, as measured by Schirmer’s test [140]. The study suggested that the hydro-alcoholic herbal extract of Nigella sativa presents a viable treatment option for dry eye disease. A novel NE formulation containing 5.5% curcuminoids has demonstrated promise as a candidate for managing dry eye in atropine-induced dry eye mice models [141]. This is demonstrated by the FDA’s approval of Restasis^®^ (Allergan, Dublin, Ireland), the European Union’s authorization of Cationorm^®^ (Santen Pharmaceutical, Osaka, Japan) for dry eye treatment, and the recent approval of Ikervis^®^—a cyclosporin A-based NE for severe keratitis [130]. Cationorm^®^, a cationic, API-free NE developed by Santen and Novagali Pharma, was introduced as a medical device in 2008 to provide extended relief from dry eye disease symptoms. In 2015, the European Medicines Agency approved Ikervis^®^, a cationic NE containing cyclosporine A (1 mg/mL), for treating severe keratitis in adult patients. Additionally, in 2022, the FDA approved the first generic version of Restasis^®^ sponsored by Viatris (Morgantown, WV, USA). Glaucoma, characterized by elevated intraocular pressure that can lead to blindness, is the second leading cause of blindness worldwide after cataracts. Traditional glaucoma treatments face challenges such as limited drug retention, poor corneal penetration, short residence time, systemic absorption, low bioavailability, lack of controlled release, poor patient compliance, and insufficient bioadhesion. Formulations from Novagali Pharma, including Novasorb^®^, Catioprost^®^, and Cationorm^®^, have advanced to clinical trials. Novasorb^®^ is a cationic NE with latanoprost as the active ingredient for glaucoma management and includes benzalkonium chloride as a cationic surfactant, enhancing adherence to the negatively charged ocular surface. Catioprost^®^, a cationic emulsion containing latanoprost, is notable for being preservative-free, unlike Novasorb^®^, which contains benzalkonium chloride, potentially causing dry eye symptoms. Cationorm^®^ and Catioprost^®^ are distinguished by their preservative-free formulas, while quaternary ammonium compounds used in emulsions can minimize preservative effects by reducing their free concentration in the aqueous phase [142]. Various NE formulations developed for different ocular disorders are depicted in Table 2.

## 8. Parenteral Nanoemulsion

NEs are ideal for loading active pharmaceutical components for parenteral drug delivery, because they provide opportunities for targeted drug delivery when used with specific ligands. Recently, research has increasingly focused on PEGylated NEs, which involve modifying droplet surfaces with polyethylene glycol (PEG) derivatives [150]. This approach aims to extend bloodstream circulation, enhance droplet surface hydrophilicity, improve steric stabilization, minimize interactions with plasma proteins and cell surfaces, and facilitate targeted delivery to tumors and inflamed areas [151].

Researchers have reported the development of a folate-based paclitaxel NE aimed at efficient tumor-targeted therapy [152]. In vitro experiments indicated that the developed NE significantly enhanced paclitaxel uptake in 4T1 breast cancer cells, exhibiting a targeting capability that was one hundred times greater than that of non-folate paclitaxel-loaded NEs. In mice with subcutaneously implanted 4T1 breast cancer, folate-based NEs improved circulation time, increased drug accumulation at tumor sites, and effectively inhibited tumor growth while demonstrating lower systemic toxicity.

A study evaluated the effectiveness of a NE (LBT-oleic acid-NE) versus a formulation containing liposome (LBT-oleic acid-Lipo) and lycobetaine (LBT) combined with oleic acid for lung cancer treatment [153]. The study also investigated PEGylated versions of these formulations. Liposomes entrapped more LBT-oleic acid than NEs, with oxygen content measurements of 31.10% for LBT-oleic acid-Lipo and 25.47% for LBT-oleic acid-NE. PEGylated lecithin was found to protect drug carriers from being recognized as foreign bodies by the mononuclear phagocytic system, reducing their removal from systemic circulation. Following 8 h, the cumulative release of LBT was lowest from LBT-oleic acid-PEG-Lipo (56.2%) compared to LBT-oleic acid-Lipo, LBT-oleic acid-PEG-NE, and LBT-oleic acid-NE. For intravenous use, emulsion droplets must be smaller (<200 nm) to prevent embolisms, in accordance with the specifications outlined in the American Pharmacopeia, USP<729>, PFAT5. Additionally, parenteral NEs can be categorized as follows: NEs designed for nutrition, NEs serving as matrices for drugs, NEs with a cationic surface charge, PEGylated NEs on their droplet surfaces, and NEs with PEGylation and targeted ligands on their droplet surfaces. It is worth noting that some individuals either have pre-existing antibodies to PEG or develop them, potentially decreasing drug effectiveness or triggering hypersensitivity reactions [154,155]. Given the extensive use of PEG in COVID-19 vaccines, the U.S. FDA now advises assessing anti-PEG antibodies in new PEG-containing drugs [156]. Most parenteral NEs for nutritional purposes utilize egg yolk phospholipids as the emulsifier, although soybean phosphatidylcholine is noted for being less susceptible to hydrolysis over shelf life. These formulations generally incorporate glycerin (2.2–2.5%) to achieve an osmolality comparable to blood, approximately 300 mOsm/kg. Additional common ingredients in approved NEs include free fatty acids such as oleic acid as co-emulsifiers, stabilizers like EDTA, and antioxidants such as α-tocopherol. Distilled water was the most commonly used aqueous phase in NEs, except in a few studies where acetate buffer, tris(hydroxymethyl) aminomethane hydrochloride buffer, or isotonic sodium chlorate solution was selected.

FDA-approved oils commonly used for parenteral administration include derivatives of soybean oil, safflower oil, MCT, olive oil, castor oil, corn oil, cottonseed oil, and glycerides of fatty acids, along with alpha-tocopherol and glycofurol. Co-surfactants that modify the surface charge of micelles can aid in crossing cell membrane barriers. Proper adjustment of surfactant and cosurfactant concentrations is crucial for creating NEs with desirable properties and incorporating bioactive substances. However, caution is necessary, as high concentrations of these agents can be clinically risky due to their potential toxicity [157].

Some studies have used marketed NEs to modify the pharmacological profiles of drugs or diagnostics. For example, pretreatment with intralipid increased the biological half-life of nano- and micron-sized superparamagnetic iron oxide particles because lipid pretreatment reduces the mononuclear phagocyte system’s capacity for uptake [158]. Encapsulating lipophilic drugs in O/W emulsions effectively resolves solubility challenges. Sorafenib, a poorly water-soluble drug, was formulated as an O/W NE for intravenous delivery using a high-energy emulsification technique [159]. The formulation, optimized via response surface methodology, included 3% MCT, 2.5% lecithin, and 1.22% polysorbate 80. This resulted in an NE with a particle size of 43.17 nm, a PDI of 0.22, and a zeta potential of −38.8 mV, making it suitable for parenteral administration in breast cancer treatment due to its dose-dependent toxicity. It has been noted that the lipophilicity of a drug in a lipid NE is crucial for its biodistribution. A higher log P, reflecting greater lipophilicity, is advantageous as it facilitates slower systemic distribution. Enhancing lipophilicity through esterification with fatty acid esters can significantly prolong the drug’s circulation time and improve its biodistribution.

A study investigated the incorporation of ciprofloxacin into Clinoleic^®^ and Intralipid^®^ total parenteral NEs, assessing their physicochemical characteristics and stability under varying drug concentrations and storage conditions [160]. It was observed that incorporating the drug did not significantly alter the droplet size, PDI, zeta potential, or pH of the NEs. All formulations exhibited high drug entrapment efficiency and sustained drug release over 24 h. When stored at 4 °C, both Clinoleic^®^ and Intralipid^®^ formulations remained stable for 180 days, maintaining consistent physicochemical properties. At room temperature, stability was retained for 60 days, with only minor pH variations. Overall, the study demonstrated successful ciprofloxacin loading into these NEs, highlighting their stability and favorable characteristics over a six-month period at 4 °C.

Additionally, the co-encapsulation of paclitaxel (PTX) with baicalein in an NE reduced PTX resistance, as baicalein potentially inhibits P-gp activity and induces oxidative stress. This NE showed greater cytotoxicity toward MCF-7/Tax cells compared to free PTX due to the combined effects of PTX and baicalein. Enhanced cellular uptake in MCF-7/Tax cells was likely due to NE-mediated endocytosis and P-gp inhibition by baicalein. Furthermore, baicalein increased intracellular reactive oxygen species production, contributing to the synergistic effects of PTX and baicalein. The NE also reduced intracellular glutathione levels and increased caspase-3 activity, leading to increased apoptosis. These findings suggest that the PTX/baicalein NE could serve as a potential agent for overcoming drug resistance, addressing paclitaxel’s limited solubility that restricts its therapeutic use.

Researchers also developed a novel NE, PTX-loaded hyaluronan solid NE, to assess its effectiveness in improving tumor targeting. The developed NE demonstrated enhanced cytotoxicity due to the extended presence of paclitaxel in the bloodstream, which led to increased accumulation in ovarian cancer cells and more effective suppression of tumor growth [161]. Intravenous NEs represent a promising delivery system for particularly lipophilic active pharmaceutical ingredients with diverse therapeutic activities. Drug delivery systems that are NE-based can reduce acute and long-term toxicity, leading to myelosuppression and tissue toxicity, compared to conventional injections currently available. Emulsifiers, oils, and other additives in NE formulations can modify their pharmacokinetic properties. Injectable lipid NEs, historically considered safe for clinical use in parenteral nutrition, have recently been proposed for drug delivery. Several formulations have already been introduced to the market, serving as carriers for nonsteroidal anti-inflammatory drugs, anesthetics, hypnotics, and corticosteroids [162].

Due to their extended circulation time, targeting ability, and immune evasion, cell membrane fragments derived from various sources like red blood cells, cancer cells, immune cells, and platelets have been used to encapsulate injectable NEs through a simple co-extrusion process using syringe filters with a pore size of < 220 nm [163,164]. Treating high-grade gliomas presents significant challenges due to the rigid blood-brain barrier that hampers efficient chemotherapy. Nonetheless, the formation of a leaky blood-brain tumor barrier creates an opportunity for targeted drug delivery using nanocarriers. By encapsulating injectable NEs with glioma cell membrane fragments, researchers have leveraged their homotypic targeting ability to deliver various chemotherapeutics as hydrophobic ion pairs. Assays on 2D and 3D cell models have shown that this method serves as a versatile drug delivery platform with chemotactic properties directed toward glioma cells. The interaction between the target cells and cell membrane fragments suggests promising translational potential for personalized nanomedicine in the near future.

The challenges of low delivery efficiency and short blood circulation of magnetic nanoparticles to malignancies by intravenous injection were effectively overcome by developing biomimetic NEs [165]. This was accomplished by encasing imiquimod, iron oxide nanoparticles, and perfluorohexane inside the membrane of a red blood cell. These NEs rapidly delivered their payload to the tumor site through magnetic targeting. Drug release was then triggered by the phase transition of perfluorohexane under ultrasound stimulation and subsequent microstreaming, which facilitated iron oxide nanoparticles’ easier penetration into the tumor tissue. The variety of intravenous emulsifiers is steadily increasing, which could allow the administration of substances via the intravenous route that were previously unsuitable due to their physicochemical properties. Recent studies on parenteral NEs are summarized in Table 3.

## 9. In Vitro Characterization Techniques for Nanoemulsions

Assessing the size distribution, average particle size, and PDI is essential for verifying dispersion homogeneity. Dynamic light scattering is the preferred method for size characterization due to its widespread availability, accuracy, and ease of use. Because of the challenges in evaporating the oil phase, O/W NEs are generally unsuitable for imaging with transmission electron microscopy or atomic force microscopy, which are typically used to visualize surface morphology and internal structure. For NEs, using a low-power electron beam (70 kV) can evade the need for staining agents, thus preventing oil vaporization during measurement. Cryogenic transmission electron microscopy is a specialized method that complements transmission microscopy, making it well-suited for examining water-containing samples. The particle charge, derived from various ionized components on the surfaces of NE droplets, plays a critical role in determining the stability and functional activity of the emulsions [172,173]. Laser-Doppler electrophoresis, also known as electrophoretic light scattering, enables the simultaneous measurement of particle size and ζ-potential using the same device [174]. NEs can exhibit rheological properties ranging from fluid to gel-like states, influenced by factors such as the volume fraction of the dispersed phase, droplet size, and the inclusion of viscosity-enhancing agents in the continuous phase [108]. Oscillatory rheology is used to evaluate the type and characteristics of NE-based gels, which are affected by droplet size and storage duration [175].

When characterizing in vitro, the first step is to evaluate transparency and distinguish between NEs and microemulsions using the light transmittance method or visual inspection. This method also helps identify potential physical instability issues that might occur during thermodynamic stability studies, processing, or storage [119]. A particle size distribution analysis should be conducted following ICH guidelines to evaluate the formulation’s physical stability under different storage conditions. Drug release from an NE is influenced by the formulation’s components, the properties of the active pharmaceutical ingredients, and the surrounding environment. The process starts with the drug migrating from the oil phase to the surfactant layer, followed by its transfer into the aqueous phase. As the drug dissolves and moves into the surrounding water, nanoprecipitation occurs, which significantly increases the drug’s surface area. This increased surface area facilitates the drug’s disintegration, a phenomenon described by the Noye–Whitney equation. In vitro drug release studies using artificial cellulose membranes with specific molecular cutoffs are conducted to determine the drug release pattern. To assess drug release from an NE, separation techniques such as dialysis, filtration, or centrifugation should be utilized to isolate the free drug from the formulation. The choice of analytical techniques will be based on the drug’s properties and the potential interferences from excipients in blank and control experiments. Drug release from orally administered NEs is evaluated by sequentially exposing them to simulated gastric and intestinal fluids, which contain bile salts and digestive enzymes. This approach is used because of the fatty acids present in the oily core of the NEs [176,177]. Ex vivo drug permeation refers to the study of how a drug encapsulated within the nanoglobules permeates or penetrates through biological tissues or barriers such as the intestinal mucosa for oral and cornea for ocular NE, which have been removed from a living organism, and are maintained under laboratory conditions. This type of study is critical in pharmaceutical research to understand the drug’s absorption characteristics and its potential effectiveness and safety. Pharmacokinetic evaluations can measure the absolute or relative drug availability and elimination from the target tissue site. For sterile formulations such as parenteral and ophthalmic, maintaining an ideal pH and osmolarity is essential to prevent tissue irritation, preserve tissue integrity, and ensure clinical performance. The tolerability of the developed ocular NEs can be assessed using the human SV40 immortalized corneal epithelial cell line (CRL-11135), human corneal epithelium-2 (HCE-2), and retinal pigment epithelial cells (ARPE-19) by examining cellular morphology, cytotoxicity, and inflammatory responses [178]. Cellular toxicity of NEs is evaluated by incubating the formulation with cultured selected cell lines and assessing cell viability either by measuring lactate dehydrogenase release or by evaluating mitochondrial dehydrogenase activity via the MTT assay [179]. To assess the toxicity of compounds absorbed through the small intestine, the Caco-2 cell model is commonly used [180]. Additionally, transepithelial electrical resistance measurements and the evaluation of tight junction protein expression in IPEC-J2 cells can be used to assess intestinal permeability and the integrity of epithelial tight junctions [181]. Higher concentrations of ethanol have potentially toxic effects, likely by compromising the cell membrane’s integrity [182]. While Solutol^®^ HS 15 and Tween 80 are approved for parenteral use, their significant hemolytic activity at higher plasma concentrations should be considered when optimizing the composition [183]. NEs contain lipid nanoparticles that could exhibit potential toxicity due to their small size, charge, and digestibility through various mechanisms [184].

As per the protocols outlined in USP Chapter <51> and ISO 11930 [185], NE antimicrobial efficacy is assessed by growing the viable microorganisms in appropriate media after being incubated with possible pathogens at prescribed concentrations. To assess the potential irritancy of an NE or its components on target tissues, a sensitivity test based on the Draize method is commonly employed. The NE can be sterilized by membrane filtration in an aseptic environment or by exposure to moist heat. Table 4 summarizes the many characterization procedures frequently used to assess NEs.

## 10. Physical and Thermodynamic Stability Studies

A stable NE maintains uniformly dispersed globules throughout its shelf life, avoiding phase separation and microbial contamination. It retains its elegance in terms of organoleptic properties and consistency. Chemical instabilities, such as oil rancidity and emulsifier hydrolysis, can be minimized by adding preservatives and antioxidants. Physical instabilities arise from interactions between the drug and excipients, leading to flocculation or aggregation of globules, which can be minimized by creating uniformly sized globules and adding ionic emulsifying agents or electrolytes. Coalescence occurs when dispersed globules combine to form larger droplets, resulting in an increase in globule size and a decrease in the number of globules.

Coalescence and Ostwald ripening are significant instability issues in emulsions, often leading to phase separation. Ostwald ripening arises in emulsions when there is a chemical potential difference between the droplets or differences in droplet curvature radii. This causes mass transfer from smaller to larger droplets with polydispersed droplets [197]. This process can be slowed or halted in W/O emulsions by adding electrolytes to the dispersed aqueous phase. In O/W NEs, the rate of Ostwald ripening can be reduced by adding a third component with lower solubility than the oil phase or using thickening agents like Aerosil COK84 [198]. The incorporation of MCT compared to long-chain triglycerides can also prevent Ostwald ripening [199].

In a recent study, O/W NEs resistant to Ostwald ripening were created by simply mixing a water phase with an oil phase that includes fatty alcohol polypropylene polyoxyethylene ether. The study concluded that the primary mechanism for droplet size growth in a spontaneously formed O/W NE is a higher concentration of surfactant, which is dependent on micelles in the aqueous phase and the oil’s high hydrophilicity [200]. The stability of lipid emulsions containing local anesthetic or analgesic drugs can be enhanced by incorporating hydrophobic excipients with lower solubility than the dispersed phase. By making the continuous phase more viscous, polymeric emulsifiers stabilize emulsions and lessen Ostwald ripening. Emulsions formulated with oils that are immiscible with water, such as mineral oil, generally show less Ostwald ripening and are easier to emulsify compared to the more miscible vegetable oils used in parenteral preparations. These destabilization processes are interrelated and can impact one another. Evaluating physical stability involves droplet size analysis using dynamic light scattering or electron microscopy, zeta potential measurements, viscosity assessments, and visual inspections for phase separation, creaming, or precipitation.

Thermodynamic stability studies are crucial for ensuring the efficacy and longevity of NEs in various applications, such as drug delivery, cosmetics, and food products. These studies evaluate an NE’s ability to maintain its structure and composition over time under different environmental conditions, ensuring it remains homogeneous without phase separation, creaming, flocculation, or coalescence. Factors influencing stability include the choice of surfactant and cosurfactant, their concentration ratios, and the method of preparation. Stress tests like heating-cooling cycles, centrifugation, and freeze-thaw cycles assess the robustness of the NE, helping to understand its kinetic stability and predict its shelf life. Techniques such as droplet size analysis, zeta potential measurements, and visual observations monitor any changes in the NE characteristics. The International Council for Harmonisation of Technical Requirements for Pharmaceuticals for Human Use guidelines, particularly Q1A (R2) on stability testing, state that these studies ensure the consistency, safety, and efficacy of pharmaceutical NEs by evaluating their ability to maintain physical, chemical, microbiological, and functional properties over time [201,202]. Stability studies involve subjecting NEs to various stress tests, including accelerated stability testing at elevated temperatures (e.g., 40 °C ± 2 °C), long-term stability testing at recommended storage temperatures (e.g., 25 °C ± 2 °C), controlled humidity levels (e.g., 75% RH ± 5%), light exposure, freeze-thaw cycles, and high-speed centrifugation to assess thermal stability, photodegradation sensitivity, and stability under extreme temperature fluctuations and gravitational forces [203]. In formulation development, NEs are first tested through six heating-cooling cycles ranging from 4 °C to 40 °C. Stable formulations are then centrifuged at 5000 rpm for 30 min to detect any instability. Finally, the stable samples undergo three freeze-thaw cycles between −21 °C and 25 °C, after which they are evaluated for dispersibility [204]. Chemical stability is analyzed using techniques like high-performance liquid chromatography to detect degradation products, while microbial tests ensure sterility and acceptable preservative concentration [205]. Packaging interactions are also studied to prevent adverse reactions. Results from these tests establish the NE shelf life, recommended storage conditions, and potential expiration date, ensuring safety, effectiveness, and high quality throughout its intended shelf life.

## 11. Future Perspectives and Advances

NEs have proven substantial potential in improving the oral bioavailability of weakly water-soluble medicines. Recent advancements are aimed at enhancing stability, achieving controlled release, and enabling targeted delivery. Key innovations include the development of mucoadhesive NEs, which adhere to the gastrointestinal tract for prolonged drug release and, therefore, extended therapeutic effects. For instance, chitosan-coated Gen-NEs have demonstrated stronger cytotoxic activity against human pharyngeal squamous cell carcinoma and significant anticancer efficacy against oropharyngeal carcinomas [206]. pH-sensitive NEs that release drugs in response to specific gastrointestinal environments are a promising approach to treat various GI diseases. Recently, the development of pH-responsive sodium alginate hydrogel-coated NEs (CUR/EMO NE@SA) for the co-delivery of curcumin and emodin has been reported. These NEs aim to provide regulated drug release and particularly target colon macrophages for the efficient treatment of inflammatory bowel disease [207]. In a similar manner, a biocompatible composite hydrogel, made from poly(L-lysine isophthalamide) and L-cystine dimethyl ester dihydrochloride, features pH/redox-dual responsiveness and is embedded with NEs, showing potential for controlled intestinal release of magnesium and other ions [208]. Furthermore, encapsulating drugs in NEs can protect them from the harsh conditions of the digestive system, improving therapeutic efficacy [209]. In ophthalmic applications, NEs offer advantages such as enhanced drug solubility, increased corneal permeability, and sustained drug release. Progress is being made in developing biocompatible and non-irritating formulations that enhance drug delivery to both the anterior and posterior chambers of the eye. Techniques like iontophoresis, where a small electric current drives the drug-loaded NE into ocular tissues, and the use of biodegradable polymers for sustained release are at the forefront of research [144]. Additionally, targeting specific ocular tissues with functionalized NEs is a growing area of interest. For parenteral administration, NEs provide a means to deliver hydrophobic drugs intravenously with improved solubility and reduced toxicity [210]. Recent studies focus on designing NEs with long circulation times and targeted delivery to specific tissues or tumors, utilizing ligands and antibodies for active targeting [211]. Advances in surface modification of NEs with PEG (PEGylation) have been crucial in reducing immune clearance and enhancing drug accumulation in target tissues. Moreover, combining NEs with other delivery systems like liposomes and nanoparticles is being explored to enhance therapeutic outcomes [212].

NE technology, while beneficial for drug delivery, faces several limitations, including stability issues such as Ostwald ripening and coalescence, high production costs, and challenges in scalability. The small droplet size restricts drug load capacity, and regulatory hurdles can complicate approval processes. Additionally, safety concerns regarding surfactants and varying effectiveness by delivery route further complicate their use. Their complex formulations and shorter shelf life also pose challenges, potentially hindering broader adoption. These limitations should be thoroughly considered when developing and implementing NE formulations for specific applications.

Ongoing and completed clinical trials, as summarized in the accompanying table (Table 5), demonstrate the growing interest in translating these formulations from the laboratory to the clinic. These trials explore various therapeutic applications, highlighting the versatility and potential of NEs in addressing unmet medical needs. Additionally, a compilation of recently filed patents, also presented in Table 6, underscores the innovative approaches being pursued in this field. These innovations include novel formulations, improved stability, and targeted delivery systems, which are expected to further advance the clinical use of NEs. The future of NEs in clinical practice looks promising, with ongoing research likely to expand their therapeutic applications and improve patient outcomes.

## 12. Conclusions

The future of NEs in drug delivery is bright, with ongoing research and technological advancements paving the way for more efficient and targeted therapies. By addressing current limitations such as stability, biocompatibility, and targeted delivery, NEs are set to play a pivotal role in the next generation of pharmaceutical formulations for oral, ophthalmic, and parenteral administration.

## Figures and Tables

**Table 2:** An overview of various nanoemulsion formulations developed for different ocular disorders.

Drug	Ingredients	Preparation Method	Characterization	In Vitro–In Vivo Findings	Disease	Reference
Brimonidine tartrate	Castor oil (oil), Acrysol K-140 and PEG 400 (surfactant mixture)	Spontaneous emulsification technique	Optimized, transparent (Grade A) SNEDDS formulation comprised 10% castor oil, 10% Acrysol K-140, and 20% PEG 400. SNEDDS formulations demonstrated a pH range of 6.8–7.4, viscosity ranging from 2.76 to 3.99 cps, and drug content ranging from 99.55 ± 0.83% to 102.14 ± 1.82%. In situ gels had a pH range of 7 to 7.5, with viscosities between 30 and 290 Pa.s, and the drug content across all batches ranged from 90% to 100%.	The self-NE system demonstrated controlled drug release for up to 8 h, with an optimum drug content of 100.25%, cumulative drug release of 92.46% up to 12 h, and followed Higuchi kinetics. The optimized in situ gel exhibited sustained drug release for 12 h and also followed Higuchi kinetics. In vitro ocular irritancy testing confirmed its suitability for ocular administration, while stability studies revealed no significant changes during storage.	Glaucoma	[143]
Ciprofloxacin (CIP)	Oleic acid and Labrafac Lipophile^®^ (oil), Tween^®^ 80 and Poloxamer 188 (surfactants)	Hot homogenization and ultrasonication	Optimized formulation (CIP-O-NE-30) showed a globule size of 121.6 ± 1.5 nm, a PDI of 0.13 ± 0.01, and a ZP of −35.1 ± 2.1 mV, with a drug content of 100.1 ± 2.0%. At ambient temperature (25 °C) and chilled (4 °C), the autoclaved CIP-O-NE-30 formulation held its consistency for one month. Surface morphology of the CIP-O-NE-30 formulation, analyzed using TEM, exhibited spherical droplets and nanoscale size.	In vitro cumulative percentage drug release in isotonic phosphate buffer (pH 7.4) over 24 h was 99.5 ± 4.5% and 86.0 ± 3.2% from control and CIP-O-NE-30, respectively. Ex vivo transcorneal permeability and flux data obtained from rabbit corneal membranes demonstrated sustained release and a 2.1-fold increase in penetration of CIP-O-NE-30 compared to the commercial CIP ophthalmic solution.	Bacterial keratitis	[144]
Dexamethasone (DEX)	Castor oil (oil), Tween 80/Carbopol 980 (surfactant mixture)	Microfluidization technique	The Plackett-Burman Design was used to screen independent variables and assess their effects on the critical quality attributes. The optimized DEX NE obtained via Box-Behnken Design exhibited a particle size of 181 ± 90 nm with a PDI of 0.36, ZP of −21.03 ± 1.68 mV, and a viscosity of 19.99 cP. The contact angle of DEX NE was 35.3° and the surface tension was 39.71 mN/m, close to the normal tear film.	In vitro release studies of plain DEX solution and optimized DEX NE utilizing the dialysis technique in simulated tear fluid showed immediate release over 3 h, followed by a slow and steady release (57 ± 2.79%) over 8 h, with a maximum release (92.93 ± 1.01%) over 48 h. In vitro cytotoxicity assays using the Statens Serum Institute Rabbit Cornea cell line confirmed good cell viability.	Inflammatory conditions	[145]
Fluconazole (FLZ)	Oleic acid (oil)/Kolliphor EL, Tween 80, Tween 20 (surfactants)/Propylene glycol, and PEG 200 (cosurfactants)	High-pressure homogenization	FLZ-loaded (0.3% *w*/*v*) sterilizable NE had droplet sizes ranging from 80.63 to 129.68 nm, PDI values < 0.25, ZP values ranging from −16.96 to −34.81 mV, pH between 3.79 and 4.78, osmolality ranging from 20.67 to >2000 mOsm/kg, and surface tension values between 34.38 and 37.43 mN/m. Higher HLB values of surfactants were found to reduce formulation stability in the order of Kolliphor EL > Tween 80 > Tween 20. NMR spectra indicated that higher molecular size offered greater stability in the order of Kolliphor EL (~2500 g/mol) > Tween 80 (~1310 g/mol). Propylene glycol (76.09 g/mol) as a cosurfactant provided better stability than PEG 200 (190–210 g/mol).	In vitro release studies indicated that the optimized FLZ NEs exhibit satisfactory sustained drug release compared to the total drug release from the reference solution at 5 h. In vitro antifungal activity tests conducted on the longest stable NEs with Kolliphor as a surfactant demonstrated inhibition zones for C. albicans, C. parapsilosis, C. glabrata, and C. tropicalis, ranging from 34 ± 4 to 36 ± 5 mm, 30 ± 2 to 34 ± 1 mm, 14 ± 2 to 17 ± 1 mm, and 34 ± 5 to 37 ± 2.5 mm, respectively, similar to the FLZ solution (*p* > 0.05).	Fungal infections	[146]
Tacrolimus	Medium-chain triglycerides (oil), Tyloxapol (surfactant)	High-pressure homogenization	Optimized cationic NE (CNE-F4) had a nanoscale particle size of 197.4 nm, a PDI of 0.119, a ZP of −0.346, an osmolarity of 278.7 mOsmol/kg, and a pH of 7.15. F3 (61.31 Pa) and F4 (59.99 Pa) demonstrated much higher mucoadhesion compared to F1 and F2, indicating stronger macromolecular interactions in F3 and F4. TEM micrographs revealed spherical, well-dispersed NE droplets. F4 demonstrated significantly higher mucoadhesion, indicating stronger macromolecular interactions. Stability data collected over a two-month period showed no significant changes in F4, indicating that the formulation remained stable.	In vitro release of F4 was less than 20% over 12 h, likely due to hindrance from the emulsion’s oil phase and gel formation. In vivo ocular surface retention study indicated that the precorneal residence time of CNE in an in situ gel (CNE-ISG) containing 0.05% tacrolimus was 50 min, significantly longer than the 25 min observed for CNE at the same concentration and 10 min for the commercial Talymus^®^ suspension (0.1% tacrolimus). Rabbits with dry eye syndrome were given 0.05% CNE-ISG (twice daily), 0.05% CNE, and 0.1% Talymus^®^ suspension (thrice daily) as treatments. Schirmer’s tear secretion test results after five days revealed no discernible change between the CNE-ISG group and the healthy group. In the CNE-ISG group, the Tear Ferning Test revealed a reversion to typical tear-fern-like crystal branches. The HET-CAM stimulation test showed no ocular irritation in the CNE-ISG group, while histological analysis showed a significant increase in goblet cells.	Dry eye disease	[147]
Tamoxifen (TAM)	Oleic acid (oil)/Tween 80 or Tween 20 (surfactants), PEG 400 (cosurfactant)	Spontaneous emulsification technique	Optimized NE (TAMNE-1) exhibited a particle size of 140.20 ± 1.50 nm, a surface charge of −27.86 ± 1.13 mV, and a PDI of 0.20 ± 0.00. The TEM image of TAMNE-1 showed spherical NE particles with a uniform size distribution. Thermodynamic stability studies (freeze-thaw cycles, heating-cooling cycles, and centrifugation) revealed high stability for both TAMNE-1 and TAMNE-1 gel.	In vitro studies showed that TAMNE-1 in a 0.3% *w*/*v* gellan gum-based gel provided a prolonged and sustained release compared to both the TAM solution and TAMNE-1. TAM was successfully radiolabeled with ^131^I to assess the in vivo efficacy of the TAMNE-1 gel. The developed gel system was found to be non-irritating and safe after histopathological assessment, with in vivo biodistribution studies showing better retention compared to plain TAM eye drops.	Photoreceptor degeneration disorder	[148]
Thymoquinone (TMQ)	Kolliphor^®^ RH40 (oil)/Capmul^®^ PG-8 NF (surfactant)	Phase inversion	TMQ-NE exhibited a particle size of 29.10 ± 0.87 nm, PDI of 0.096 ± 0.03, pH value of 6.2 ± 0.2, TMQ content of 99.56 ± 0.43%, and osmolarity of 302 ± 1.5 mOsm/kg. TEM micrographs of the TMQ-loaded NE displayed spherical oil globules with smooth edges and showed no signs of coalescence or agglomeration. Thermodynamic stability tests clearly indicated no signs of phase separation or coalescence.	In vitro drug release studies demonstrated that TMQ-NE released 84.87 ± 0.18% of TMQ in 10 h, and the ex vivo corneal permeability study indicated approximately 90% permeation. Safety assessments, including the ex vivo HET-CAM assay, in vivo Draize test, and histological assessment, confirmed its suitability for ocular administration. In vivo pharmacodynamic evaluation conducted in a rabbit model showed that the optimized TMQ-NE significantly reduced intraocular pressure by 39.59 ± 0.88% within 1.5 h post-administration.	Glaucoma	[149]
Travoprost	Labrafac Lipophile^®^ (oil), Tween 80 (surfactant)	Emulsion inversion point	Optimized Travoprost NE (F3), which includes 1% *w*/*v* oil and 2% *w*/*v* Tween 80, demonstrated a globule size of 157.4 ± 0.36 nm, PDI of 0.56 ± 0.00, and ZP of −16.6 ± 0.38 mV, with a drug content of 99.51 ± 0.12%. Accelerated stability studies of F3 conducted at 25 °C ± 2 and 60% ± 5% relative humidity over three months indicated no significant change in the PDI value (*p* > 0.05) due to steric stabilization. Morphological studies revealed spherical droplets without aggregates, and the recommended sterilization method after evaluation was membrane filtration.	In vitro drug release studies conducted using a dialysis-based method demonstrated a sustained release pattern for travoprost, in contrast to the immediate release observed with Travatan^®^ drops. Higher C_max_ and AUC values noted in the in vivo pharmacokinetic study in rabbits supported the sustained intraocular pressure-lowering action, or pharmacodynamic effect, of the travoprost NE, indicating superior ocular bioavailability compared to Travatan^®^ eye drops. Results from the ocular tolerability study indicated that both the selected F3 NE and Travatan^®^ eye drops were non-irritating to the eyes of the rabbits.	Glaucoma	[135]

**Table 3:** Active pharmaceutical ingredient(s), key formulation components, preparation methods, therapeutic conditions, characterization, and in vitro–in vivo findings of parenteral nanoemulsions.

Active(s)	Inactive Ingredients	Method	Condition	Characterization	In Vitro–In Vivo Findings	Reference
Amphotericin B (AmB)	Kolliphor^®^ HS15, Brij^®^ 52, isopropyl myristate	Ultrasonication	Visceral leishmaniasis	Optimized NE (NE5) exhibited an ideal droplet size (31.2 ± 0.4 nm), PDI (0.241 ± 0.009), and ZP (−14.60 ± 3.35 mV). The drug-loaded NE (AmB-NE5) had a droplet size of 49.2 ± 0.7 nm, PDI of 0.317 ± 0.002, and ZP of −16.14 ± 2.91 mV. Both NE5 and AmB-NE5 formulations had a neutral pH (6.0–7.5) ideal for parenteral therapy. AmB did not alter the system’s thermal behavior, likely due to dispersion within the internal phase.	Leishmanicidal activity of the NE was assessed by counting the total number of live parasites after 6 h of incubation. In vitro antileishmanial activity, demonstrated by EC_50_ values, showed that AmB-NE5 was comparable to the commercial micellar AmB formulation (Anforicin^®^).	[166]
Gliclazide	Olive oil, egg lecithin	Hot homogenization followed by ultrasonication	Diabetes mellitus	Radiolabeled NEs had an average droplet size of 24.7 nm, PDI of 0.21, ZP of −45.3 mV, pH of 7.4, osmolality of 285.3 mOsm/kg, viscosity of 1.24 mPa.s, and a high radiochemical yield of 99.3 ± 1.1%.	In silico analysis indicated that the labeling did not affect the biological activity of gliclazide. After i.v. administration of the NE, pancreas uptake was highest in normal rats, with 19.57 ± 1.16% at 1 h and 12 ± 0.13% of the injected dose at 4 h. In contrast, diabetic rats had 8.51 ± 0.16% at 1 h and 5 ± 0.13% at 4 h, suggesting the potential of radioiodinated gliclazide NE as a tracer for pancreatic β-cells.	[167]
Inducible T cell co-stimulator-fragment crystallizable region (ICOS-Fc), Sorafenib (SOR), Temozolomide (TMZ)	Soybean oil, Egg yolk phospholipids, Glycerin	Stepwise addition and mixing with Intralipid^®^ emulsion	Malignant melanoma	Intralipid^®^-based formulations had particle sizes ranging from 244.6 ± 18.0 to 348.3 ± 6.1 nm, PDI from 0.005 to 0.146, ZP values from −15.72 ± 1.83 to −39.52 ± 7.22 mV, and recovery rates from 68 ± 5.0% to 116 ± 10.2%. The drug combination was effectively incorporated into the lipid matrix without significant alterations in the mean size of the intralipid droplets.	The drug combination in the intralipid emulsion inhibited tumor growth and angiogenesis by engaging the immune system, with ICOS-Fc being a key component. Sub-therapeutic doses of the drugs effectively decreased tumor development in subcutaneous melanoma mouse models, likely due to the NE’s targeting properties.	[168]
Irinotecan (IRI), Doxorubicin (DOX)	Omnipaque 350^®^ (iohexol, 350 mg/mL)	Tessari technique	Metastatic colorectal cancer	Particle size of the O/W emulsion for DOX ranged from 107.2 nm to 314.2 nm, while the W/O ranged from 162.8 nm to 167.2 nm (*p* = 0.49). For IRI, the O/W had smaller particle sizes (28.9 nm to 56.4 nm) compared to the W/O emulsion (141.8 nm to 149.8 nm) (*p* < 0.0001). The IRI-lipiodol emulsion demonstrated optimal stability in an acidic environment, with particle sizes from 79 nm to 207 nm, whereas the DOX-lipiodol emulsion was most stable at pH 10.4, with smaller particle sizes from 36.6 nm to 98.5 nm over time.	Compared to the drug alone, the AUC for IRI was significantly lower after portal venous chemoembolization using IRI-lipiodol (131 mg·min/mL vs. 316 mg·min/mL). IRI-lipiodol NEs retained the drug in the liver through first-pass extraction. In tumor-bearing WAG/Rjj rats, biodistribution studies showed higher drug concentrations in tumors following transarterial chemoembolization (29.19 ± 12.33 mg/g) compared to portal venous chemoembolization (3.42 ± 1.62 mg/g) or i.v. administration (1.05 ± 0.47 mg/g).	[169]
Tea tree oil (TTO)	Tween^®^ 80, Pluronic^®^ F68	Spontaneous emulsification and nanoprecipitation methods	Sepsis	TTO NEs stabilized with Pluronic 168 and lecithin had an average droplet size of 146 ± 1 nm, PDI of 0.1 ± 0.01, and ZP of −38 ± 0.6 mV. TTO nanocapsules (NCs) prepared with Eudragit RS100 had an average globule size of 197 ± 0.2 nm, PDI of 0.1 ± 0.1, and ZP of +33 ± 0.5 mV. The kinetic stability of the NEs was confirmed through stability studies at 4 °C and 25 °C. NCs exhibited greater stability against photodegradation compared to NEs.	NCs demonstrated lower cytotoxicity compared to NEs. In the cecal ligation and puncture sepsis model, both NCs and NEs diminished neutrophil infiltration in peritoneal lavage and kidneys. Additionally, they showed efficacy in reducing bacterial growth in peritoneal lavage, raised group thiols in lung and kidney tissues, and mitigated sepsis-induced damage.	[170]
XBP1 inhibitor KIRA6, α-Tocopherol	Phosphatidylcholine, medium-chain triglyceride	Emulsification followed by probe sonication	Adipose hypertrophy and hyperplasia	NEs maintained a stable spherical structure (100–150 nm) for one month at 4 °C. Drug release studies using dyes showed initial retention within the carrier, followed by a gradual release over 10 to 24 h.	Low-toxicity NEs could effectively target (pre)adipocytes through subcutaneous or systemic administration, potentially reducing adverse effects in vivo. They alleviated oxidative stress and endoplasmic reticulum stress in (pre)adipocytes, significantly suppressed aberrant adipogenic differentiation, reduced lipid droplet transfer and storage, and impeded the advancement of obesity. It reduced the risk of non-alcoholic fatty liver disease associated with obesity in obese female mice.	[171]

**Table 4:** Various characterization techniques typically utilized for oral, ocular, and parenteral nanoemulsions.

Technique	Principle	Evaluation Parameters	Reference
Percentage transmittance	The percentage transmission is calibrated to zero using a filter and to 100% using a transparent cuvette filled with water in the colorimeter. Test samples are then placed in the cuvette, and their absorbance is measured against double-distilled water as a reference at 650 nm. The percentage of transmitted light (%T) is calculated using the following equation: A = 2 − log%T, where A is absorbance and %T is transmittance percentage.	Visual clarity of the NEs.	[186]
Conductivity	The Pt/platinized electrode for conductivity measurement is immersed in the sample. While stirring, the temperature is gradually increased at a rate of 1 °C/min, and the change in conductivity is recorded in µS/cm.	Identify the type of NE and check phase inversion phenomenon.	[10]
Viscosity	A multipoint viscometer, equipped with an appropriate spindle, is employed to measure the viscosity of NEs at various RPM settings under controlled temperatures.	Influences the residence time and spreadability of the formulation.	[187]
Particle size and size distribution	Based on the intensity of scattered laser light, particle sizes can be measured.	Provides the size of developed NEs.	[188]
Particle morphology	A drop of the diluted NE is placed on a formvar-stained copper grid, left to dry completely, stained negatively with phosphotungstic acid or uranyl acetate, and analyzed using a transmission electron microscope operating at specific kV.	Evaluate the morphology of the nanodroplets.	[171]
Dilution potential	The prepared NEs are diluted tenfold with the external phase before further analysis.	The occurrence of coalescence or phase separation indicates the physical instability of the NE.	[23]
pH and osmolarity	The pH is measured using a pH meter, which has been previously calibrated with standard buffer solutions of pH 4, pH 7, and pH 9. The osmolarity of the solution is measured using an osmometer.	Indicates the pH of the NEs. The ideal homeostatic range for osmolarity is between 270 and 310 mOsmol/L.	[149]
In vitro drug release	The drug release study is conducted in a Franz diffusion cell using buffer or simulated tissue fluid as the receptor medium. A synthetic cellophane dialyzing membrane with a specific molecular weight (kDa) cutoff is used as the diffusion membrane.	The drug release mechanism is determined by fitting the data to different mathematical models. The correlation coefficient (r^2^) is used to identify the release kinetics.	[189]
Ex vivo permeation	Using the Franz diffusion cell, the NE or control is placed in the donor chamber, and simulated tissue fluid or buffer with a specific pH is added to the receiver compartment. A biological membrane is used as a barrier.	The steady-state flux, permeability coefficient, and lag time are calculated.	[190]
In vivo pharmacokinetics	NE or control is administered through a suitable route to Wistar rats. Blood samples are assayed using analytical techniques to evaluate in vivo pharmacokinetic parameters.	The drug plasma level and various pharmacokinetic parameters (T_max_, C_max_, and AUC) are assessed.	[191]
Stability and shelf life	Stress stability conditions generally include aging, temperature fluctuations, centrifugation, and agitation. For the optimized NE, stability is initially assessed daily, then on a weekly basis, evaluating parameters such as pH, coalescence, droplet size, breaking, flocculation, and precipitation.	Phase separation is accelerated by heating-cooling cycles, freeze-thaw cycles, and centrifugation tests, as demonstrated by changes in various NE parameters.	[192]
Ocular irritation test	In vivo ocular sensitivity studies are performed using the Draize technique. Approximately 60 μL of the sterile NE is administered to the eyes of albino rabbits (2–3 kg) in the treated group, while control groups receive normal saline. The formulation is applied twice daily for 21 days.	Animals are monitored for ocular sensitivity reactions, such as redness, discharge, conjunctival swelling, edema, etc.	[193]
Pyrogen test	Combine lysates from Limulus polyphemus amoebocytes with 0.1 mL of the test sample to examine for the existence of a gel clot, then incubate for an hour at 37 °C. Use the direct transfer method to inoculate the test sample directly into two sample tubes that contain culture media: soybean casein digest medium and fluid thioglycollate medium. For the membrane filtration test, pass the sample through membrane filters under vacuum. Slice the membrane in half, then place each half in a different test tube with soybean casein digest agar (for total aerobic microbial count) and Sabouraud dextrose agar (for total mixed yeasts and molds).	Compared to other methods, the Limulus lysate test provides an easy-to-use, inexpensive, dependable, sensitive, and specific way to test for endotoxins. The direct transfer method requires more skill, despite being a relatively straightforward process. However, the USP officially recognizes the membrane filtration process, which is more accurate.	[194]
Ocular cytotoxicity study	Human epithelial conjunctival cells (IOBA-NHC) are incubated with NEs for designated periods. Cytotoxicity is measured using the MTT assay. To evaluate cell death, flow cytometry is employed with annexin V labeled with fluorescein isothiocyanate and propidium iodide to distinguish between early apoptotic and late apoptotic or necrotic cells.	Estimate the cell viability and proliferation.	[195]
Ocular toxicity test	The hen’s egg test-chorioallantoic membrane (HET-CAM) is utilized to assess the irritation potential of NEs. In this test, the formulation is applied directly to the CAM of a fertilized hen’s egg. The CAM is then visually monitored for irritation reactions, such as blood coagulation, bleeding, and hyperemia.	Evaluate the irritation potential of developed formulations.	[196]

**Table 5:** Ongoing and completed clinical trials (https://clinicaltrials.gov/; accessed on 12 October 2024) of nanoemulsion-based formulations developed for oral, ocular, and parenteral delivery.

Summary	Indication/ Conditions	Active Constituent(s)	Route	Phase	Enrolment (#)	Identifier
A randomized clinical trial aims to assess whether curcumin NE influences pro-inflammatory biomarkers in plasma and breast adipose tissue. It evaluates the adherence, tolerability, and safety of two different doses of NE curcumin in women at high risk of developing breast cancer.	Breast cancer	Curcumin	Oral	Interventional	29	NCT01975363
A randomized, double-blind pilot study aims to evaluate the feasibility of using Functional Assessment of Cancer Therapy–Endocrine Symptoms scores to detect changes in symptoms and well-being in postmenopausal women with breast cancer. The study assess the effects of aromatase inhibitors after 3 months of NE curcumin treatment versus a placebo.	Joint pain in breast cancer survivors	Curcumin	Oral	Interventional	42	NCT03865992
The prospective cohort study seeks to assess symptom changes in patients with mild to moderate dry eye using the Ocular Surface Disease Index and advanced clinical instruments following the recommended dosage of the new NE eye drops, Systane Complete.	Dry eye syndrome	Propylene Glycol 0.6%	Ocular	Phase 2	30	NCT06188260
The safety of a 0.05% clobetasol propionate ophthalmic NE compared to a 1% prednisolone acetate formulation is being evaluated for managing inflammation and pain following cataract surgery in children.	Anti-inflammatory	Clobetasol Propionate	Ocular	Phase 3	60	NCT05724446
Aims to show that a topical ophthalmic NE of difluprednate 0.05% (Tolf^®^, Poen Laboratories, CABA, Argentina) offers high anti-inflammatory efficacy, making it suitable for use following cataract surgery.	Anti-inflammatory	Difluprednate 0.05%	Ocular	Phase 4	255	NCT04631315
A study compares the incidence of parenteral nutrition-associated cholestasis in newborns requiring prolonged parenteral nutrition, specifically between those receiving cyclic versus continuous parenteral nutrition.	Nutritional supplement	Lipid-based NE	Parenteral	Interventional	50	NCT02692326

**Table 6:** A compilation of recently filed patents for pharmaceutical nanoemulsions, highlights of their innovation.

Application ID	Title	Summary of Invention	Potential Bioactivities
US-20240277615-A1	Self-emulsifying composition, production methods, and uses thereof	A self-emulsifying, stable oil-in-water emulsion containing a neutral hydrophilic surfactant and a combination of hydrophobic excipients selected from glycerol monooleate, miglyol 812, and propylene glycol monocaprylate. Most droplets in the emulsion are under 180 nm, with half ranging from 90 to 120 nm.	Intranasal and intravenous delivery of statins and phenytoin
US-20240261219-A1	New oil-in-water nanoemulsion	Preparation of stable, natural oil-in-water NE based on vegetable oil, ideal for delivering fat-soluble substances. It is suitable for oral use and can be applied in pharmacology, cosmetics, and as a food additive.	Oral delivery of fat-soluble substances
US-20240261391-A1	TLR7 agonist conjugated peptide-based novel coronavirus nanoemulsion vaccine and preparation thereof	A novel coronavirus vaccine featuring a TLR7 agonist-conjugated peptide as the antigen and an oil-in-water NE with squalene as the adjuvant. The antigen is derived from the SARS-CoV-2 S protein, and the vaccine demonstrates thermal stability.	Protection against coronavirus SARS-CoV-2 wild-type and mutant strains
US-20240207235-A1	Nanoemulsion formulation for treating diabetes mellitus (DM)	Formulation of a stable NE for diabetes mellitus combines pioglitazone with Nigella sativa oil. Drug-loaded NE with Nigella oil significantly reduces blood glucose levels more effectively than standard pioglitazone formulations.	Lowering blood glucose levels in diabetes mellitus
US 20240108710 A1	Nanoemulsion adjuvant composition for pneumococcal conjugate vaccines	Stable NE adjuvant composition for pneumococcal conjugate vaccines is made up of emulsifiers (sorbitan esters), solubilizers (terpenes), surfactants, cationic lipids, or their combinations.	Prevention of pneumococcal disease
US 20240058434 A1	Nanoemulsion universal influenza vaccine	Intranasally administered NE vaccine contains a computationally optimized influenza immunogen or protein, along with an aqueous phase, pharmaceutically acceptable oil, a blend of cationic and non-ionic surfactants, and at least one pharmaceutical-grade organic solvent.	Protection against multiple strains of influenza virus
US 20240041974 A1	Nanoemulsion ophthalmic composition comprising cyclosporine and menthol, and preparation method thereof	NE ophthalmic composition composed of cyclosporine, castor oil, hydrophilic and hydrophobic emulsifiers, menthol, and an aqueous solvent. It offers excellent stability and effectively reduces ocular irritation, grittiness, and blurry vision.	Dry eye syndrome
US 20240042016 A1	Nanoemulsion vaccine compositions and methods for suppressing reactivity to multiple food allergens	The NE compositions consist of poloxamer or polysorbate surfactant, an organic solvent, a halogen-containing compound, oil, and water, along with one or more targeted food allergens.	Suppression of activity to multiple food allergens
US 20230398164 A1	Intra-oral nanoemulsion including monolayer surfactant-bound particles for balancing histamine response	Preparation of an ingestible, intra-oral NE containing monolayer surfactant-bound particles suspended in a continuous phase to balance the histamine response in subjects.	Suppression of histamine response
US 20230201117 A1	Ophthalmic nanoemulsion compositions	Sterile ophthalmic NE formulation designed for treating ocular conditions such as glaucoma. It contains brinzolamide, brimonidine, and bimatoprost, and may include an in situ gelling agent, with or without preservatives.	Glaucoma

## Data Availability

The data presented in this study is contained within this article.
